# Proteostasis Regulators in Cystic Fibrosis: Current Development and Future Perspectives

**DOI:** 10.1021/acs.jmedchem.1c01897

**Published:** 2022-04-04

**Authors:** Irene Brusa, Elvira Sondo, Federico Falchi, Nicoletta Pedemonte, Marinella Roberti, Andrea Cavalli

**Affiliations:** † Department of Pharmacy and Biotechnology, University of Bologna, 40126 Bologna, Italy; ‡ Computational & Chemical Biology, Istituto Italiano di Tecnologia, 16163 Genova, Italy; § UOC Genetica Medica, IRCCS Istituto Giannina Gaslini, 16147 Genova, Italy; ∥ Molecular Horizon srl, 06084 Bettona, Italy

## Abstract

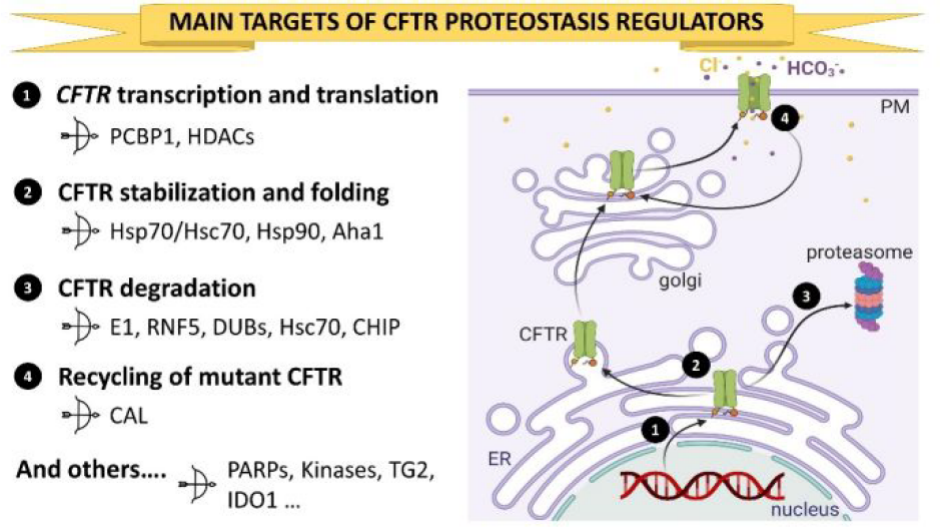

In cystic fibrosis (CF), the deletion of phenylalanine 508 (F508del) in the CF transmembrane conductance regulator (CFTR) leads to misfolding and premature degradation of the mutant protein. These defects can be targeted with pharmacological agents named potentiators and correctors. During the past years, several efforts have been devoted to develop and approve new effective molecules. However, their clinical use remains limited, as they fail to fully restore F508del-CFTR biological function. Indeed, the search for CFTR correctors with different and additive mechanisms has recently increased. Among them, drugs that modulate the CFTR proteostasis environment are particularly attractive to enhance therapy effectiveness further. This Perspective focuses on reviewing the recent progress in discovering CFTR proteostasis regulators, mainly describing the design, chemical structure, and structure–activity relationships. The opportunities, challenges, and future directions in this emerging and promising field of research are discussed, as well.

## 1 Introduction

Cystic fibrosis (CF) is a lethal genetic disease caused by defects in the cystic fibrosis transmembrane conductance regulator (CFTR), a cAMP-dependent chloride and bicarbonate ion channel that is widely expressed at the plasma membrane (PM) of several epithelial cells.^1^ CFTR is composed by two membrane spanning domains (MSDs) that form an anion-selective pore, two nucleotide binding domains (NBD1 and NBD2), which contain ATP binding sites and a regulatory region (R).^2,3^ Currently, over 2000 mutations have been identified,^4^ but to date, the pathogenicity has been confirmed only for 382 mutations.^5^ CF-causing mutations are commonly classified into six classes: (1) class I mutations result in the absence of CFTR mRNA and/or protein; (2) class II mutations cause protein misfolding and its premature degradation; (3) class III mutations impair the gating of the channel; (4) class IV mutations lead to decreased channel conductance; (5) class V mutations reduce the amount of CFTR channels at the PM; and (6) class VI mutations reduce the stability of the CFTR protein at the PM.^6,7^ The most prevalent CFTR mutation (around 80%) is the deletion of phenylalanine 508 (F508del), a class II mutation that is associated with misfolding and defective gating of the mutant protein.^8,9^ The misfolding defect results in reduced stability of F508del-CFTR, retention of the mutant channel at the endoplasmic reticulum (ER), and premature degradation by the ubiquitin–proteasome system (UPS), which causes a reduced expression of F508del-CFTR at the PM.^10−12^ The gating defect results in reduced activity of the mutant channel due to its abnormal persistence in the closed state.^13^ As a consequence, the epithelial fluid transport in the airway is dysregulated and leads to the production of a thickened mucus that favors chronic bacterial colonization, inflammation, and ultimately leads to lethal respiratory failure.^7^ Depending on the specific basic defect stemming from the CFTR mutation, distinct drugs, namely, CFTR modulators, with different mechanism of actions are necessary.^14^ CFTR modulators include correctors, potentiators, stabilizers, and amplifiers. Correctors increase the number of mutant CFTR channels at the PM acting as pharmacological chaperones or as proteostasis regulators. Pharmacological chaperones are thought to act directly on the mutant CFTR, stabilizing or improving specific domains’ interaction. Instead, proteostasis regulators target components of the CFTR regulome, such as chaperones, cochaperones, kinases, or ubiquitin ligases that affect the synthesis, folding, stability, and trafficking to the plasma membrane of the mutant channel.^15,16^ Potentiators improve channel gating of CFTR proteins expressed at the PM, directly binding to the mutant channel.^17,18^ Amplifiers increase *CFTR* mRNA translation and stimulate protein expression.^19^ Stabilizers increase the quantity of CFTR channels at the PM by anchoring the defective protein^20^ or stabilizing its interaction with other membrane components.^21^

**Figure 1:**
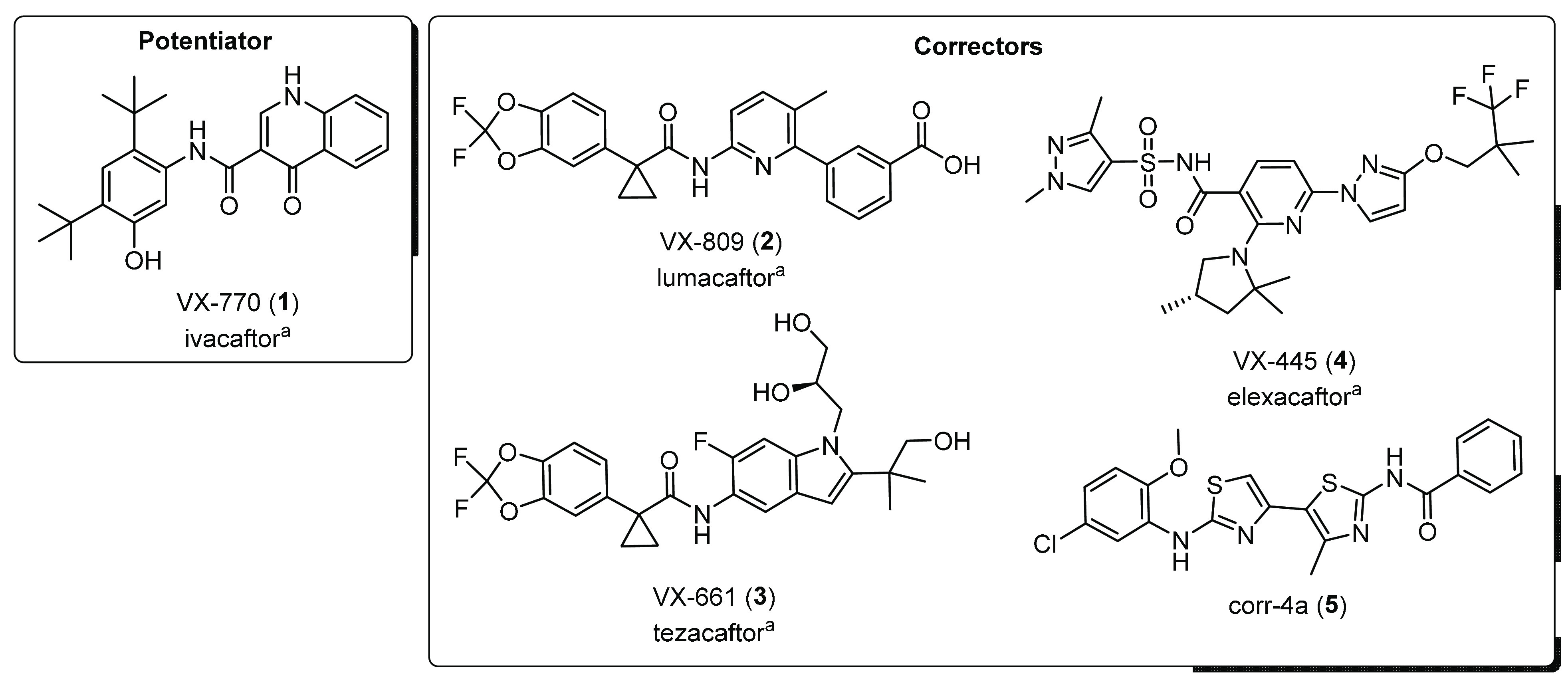
Structures of the potentiator VX-770 (**1**) and of the correctors VX-809 (**2**), VX-661 (**3**), VX-445 (**4**), and corr-4a (**5**). ^a^Approved drugs.

During the past 10 years, several efforts have been made to develop and approve new effective therapeutic strategies to restore CFTR biological function in a large cohort of CF patients. For example, ivacaftor or VX-770 (**1**, Figure 1)^22^ is a potentiator that first received marketing authorization in 2012 (with the commercial name of Kalydeco) to treat CF patients who have at least one copy of the G551D mutation, subsequently expanded to a selection of class III and IV mutations. Instead, VX-809, also known as lumacaftor (**2**, Figure 1),^23^ was the first corrector to be approved for therapeutic use in CF patients carrying the F508del mutation and, along with the potentiator **1**, constitutes the combination drug Orkambi, approved by both FDA and EMA in 2015. Many clinical studies highlighted positive results on lung function, increased body mass index (BMI), reduction of sweat Cl^–^ concentration, and lung clearance index (LCI).^24,25^ However, frequent drug intolerance and respiratory adverse effects were observed in patients treated with Orkambi.^26,27^ VX-661, also known as tezacaftor (**3**, Figure 1), is an analogue of **2** with improved pharmacokinetics and less side effects, and the **3**/**1** co-therapy (trade name Symdeko) received marketing authorization in 2018.^28^ Different clinical trials showed that the **3**/**1** combination displays effects similar to those of Orkambi in F508del homozygous patients.^29^ Notably, patients heterozygous F508del with G551D or with residual function mutations are more responsive to **3**/**1** combination than F508del homozigous.^30,31^**2** and **3** are considered first-generation correctors and act as pharmacological chaperones that stabilize the CFTR structure by improving the interdomain interactions and CFTR folding.^12,32,33^ Instead, the next-generation corrector VX-445, also known as elexacaftor (**4**, Figure 1), likely acts on different binding sites than the first-generation correctors.^34^ Indeed, VX-445 exhibits additive or synergistic effects in combination with a first-generation corrector (and with the potentiator **1**), leading to markedly increased PM expression of F508del-CFTR.^34,35^ Recent phase 3 studies by Vertex confirmed safety and benefits of **4** in CF patients who are homozygous for the F508del mutation or heterozygous for the F508del and a minimal function (MF) mutation.^35^ In the F508del cohort who was receiving standard **1**/**3** treatment, the addition of **4** resulted in 11.0 point rise in ppFEV_1_ (percent predicted forced expiratory volume in 1 s).^35^ In the MF group, treatment with **1**/**3**/**4** resulted in an increase in ppFEV_1_ of 13.8 percentage points.^36^ Strikingly, **4** is now included in a triple drug combination (trade name Trikafta in the U.S. and Kaftrio in Europe) together with **1** and **3**, for CF patients 12 years and older who have at least one F508del mutation, or another mutation known to be responsive to the drug (for the complete list of mutations, see Trikafta.com).^36,37^

Orkambi and Symdeko still represent a standard care for many CF patients, albeit their clinical effects remain moderate.^38^ Although Trikafta has undoubtedly proved to be a breakthrough in CF treatment, by significantly slowing down CF progress with substantiated clinical benefits,^39^ it fails to fully restore mutant CFTR function.^34,40^ Indeed, it has been demonstrated that the **3**/**4** combination can rescue F508del-CFTR activity only up to 65% of the wild-type (WT) CFTR activity level.^34,40^ Hence, development of new pharmacological chaperones acting with different mechanisms or with ameliorated characteristics is currently a thriving research field. Two successful examples are the recent search for optimized analogues of the bithiazole corr-4a (**5**, Figure 1)^41,42^ and the development of multitarget compounds able to simultaneously act as antiviral agents and F508del-CFTR correctors.^43,44^ For more detailed information about the promising pharmacological chaperone correctors under clinical trials or currently in study, there are several recent reviews available.^45−47^ On the other hand, no CFTR proteostasis regulator has entered the market to date. Nevertheless, the research in this field is flourishing, and many proteostasis targets have been recently uncovered due to siRNA-mediated silencing techniques, proteomic and interactomic studies,^15,48−51^ while others remain elusive yet. Indeed, the development of new compounds targeting biological components of the CFTR physiological pathway may be useful to optimize combination therapies for those patients with mutations (in particular, affecting protein maturation, trafficking, and stability at the PM), poorly responsive to current treatments.

This review summarizes the recent progress in the discovery of CFTR proteostasis regulators. In particular, we will review and discuss studies that led to discovering active compounds affecting different CFTR-related targets. Understanding their mechanism of action would facilitate structure–activity relationships (SAR) and could inspire the medicinal chemistry community to develop novel promising molecules with clinical potentiality. Likewise, the new compounds might represent effective chemical probes useful to dissect biological processes involved in CFTR dysfunctions that lead to CF.

## 2 Targeting RNA Binding Proteins

As CFTR is a monomeric polytopic membrane protein, its biosynthesis occurs at the ER and CFTR assembly and domains folding involve both co- and post-translational translocation events.^52^ CFTR polypeptide is synthesized by ribosomes present in the rough ER and, as the nascent chain emerges from the ribosome, is co-translationally translocated to the ER lumen by the cytosolic signal recognition particle (SRP).^53^ Subsequent assembly of MSDs and NBDs requires cytosolic and lumenal chaperones including Hsp70, Hsp40, Hsp90, and others.^54−56^ Once CFTR transmembrane domains are folded properly, the polypeptide associates with a complex set of cellular proteins that facilitate translocation across the ER membrane and integration into the lipid bilayer.^52^ However, the SRP-dependent co-translational translocation is reported to direct correct topology for less than half of nascent CFTR chains.^52^ Furthermore, mutations that reduce *CFTR* mRNA levels or impair CFTR translation, as well as those that lead to misfolded, unstable, or defective proteins, exacerbate inefficiencies of CFTR biosynthesis, folding, and trafficking.^57,58^

**Figure 2:**
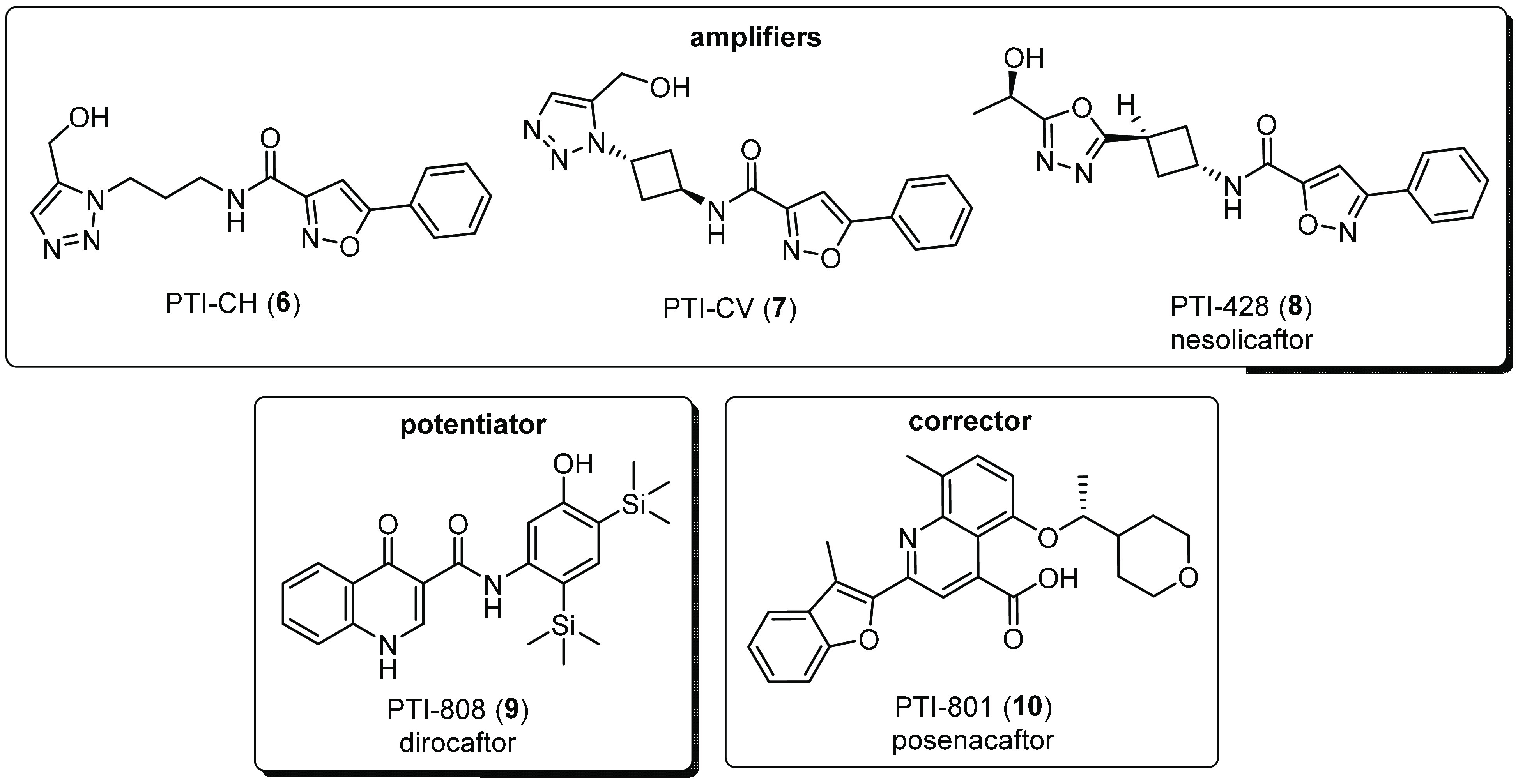
Structures of the amplifiers PTI-CH (**6**), PTI-CV (**7**), and PTI-428 (**8**) of the potentiator PTI-808 (**9**) and of the corrector PTI-801 (**10**) developed by Proteostasis Therapeutics Inc.

Several pharmaceutical companies gained interest into associations of molecules with different mechanisms. In order to identify novel classes of molecules exhibiting synergy with potentiator **1** and corrector **2**, Proteostasis Therapeutics Inc. recently performed a phenotypic high-throughput strategy (HTS) of approximately 54000 small molecules selected for novelty and drug-like properties.^59^ With this strategy, the novel class of CFTR modulators called amplifiers was identified. Indeed, the phenylisoxazole PTI-CH (**6**, Figure 2), which was selected as a representative compound of this novel class of small molecules, nearly doubled the activity of **1** and **2** when coadministered in primary human bronchial epithelial (HBE) cells. These results suggested that **6** might possess a distinct mechanism relative to known modulators. Further in vitro experiments highlighted that **6** increased CFTR protein expression across different mutations, including F508del, by increasing *CFTR* mRNA levels by ∼1.5 to ∼2-fold in HBE cells. This enhancement was specific for CFTR transcript and did not lead to induction of cytosolic or ER-associated cellular stress responses.^59^ The same year, Bear and co-workers provided the first evidence that the amplifier **6** could enhance Orkambi efficacy in nasal cultures from patients bearing the rare mutation ΔI1234_R1239.^19^ This effect was further corroborated using a CRISPR/Cas9-edited HBE cell line harboring this rare mutation. In this cell model, treatment with compound **6** increased ΔI1234_R1239-*CFTR* mRNA, and when combined with Orkambi, it significantly enhanced CFTR channel activity compared to that in Orkambi treatment alone.^19^ Afterward, Miller and co-workers investigated the mechanism through which amplifiers stabilize *CFTR* mRNA and showed that they might enhance translational efficacy by increasing *CFTR* mRNA association with polysomes.^60^ Indeed, using chemical proteomics, the authors showed that the phenylisoxazole PTI-CV (**7**, Figure 2), an analogue of **6** with better pharmacokinetic and drug-like properties, could bind to the poly-r(C) binding protein 1 (PCBP1). PCBP1 is a RNA binding protein that was reported to regulate *CFTR* mRNA levels in mouse oocytes.^61^ Notably, amplifier **7** showed an affinity for RNA-bound PCBP1 higher than that of free PCBP1,^60^ suggesting that amplifiers might increase CFTR expression through promoting translation, an innovative mechanism that is independent of the CF-causing mutation and genotype. Thanks to these outstanding results, Proteostasis Therapeutics Inc. advanced to early phase clinical trials the phenylisoxazole amplifier PTI-428, also known as nesolicaftor (**8**, Figure 2), chosen as the most promising drug candidate of the class.

Notably, the company is also developing other molecules for combination therapies: the 4-oxo-1,4-dihydroquinoline potentiator PTI-808, also known as dirocaftor (**9**, Figure 2), and the quinoline-4-carboxylic acid corrector PTI-801, also known as posenacaftor (**10**, Figure 2). The triple combination **8**/**9**/**10** increased the CFTR-dependent chloride secretion to almost normal levels in F508del-expressing cells. Furthermore, in a phase 1/2 study (NCT03500263), this triple combination regimen has an acceptable safety and tolerability profile and led to a statistically significant reduction in sweat chloride concentration and improvement in lung function (8% in ppFEV_1_) compared to placebo in F508del homozygous patients.^62^ Currently, a phase 1/2 clinical trial is assessing the triple combination treatments’ effectiveness in 180 patients either homozygous for the F508del or heterozygous for the F508del CFTR genotype, treated for a longer period (28 days, NCT03251092). These modulator drugs were also included in a HIT-CF project in February 2019 with the purpose of testing **8**, **9**, and **10** in intestinal organoids of patients carrying rare CF genotypes.^63^ No results have been published yet. Other early stage clinical trials are currently ongoing to assess the safety and efficacy of **8** in CF patients on stable treatment with Kalydeco (NCT03258424), Orkambi (NCT02718495), and Symdeko (NCT03591094). Just early results from the phase 2 clinical trial on 24 CF patients (ages ≥18 years) homozygous for F508del and receiving background Orkambi therapy were released. Treatment for 28 days with daily doses of 50 mg of **8** or placebo, followed by a 7 day follow-up period, caused an increase by 5.2% points over days 14–28 in the ppFEV_1_ of the treated ones, and the therapy was well-tolerated.^64^ Additional data are expected shortly. However, recent in vitro investigations by Galietta and co-workers showed that **8**, as expected, was effective in improving the rescue of F508del-CFTR but failed to increase the rescue of other CFTR mutants, such as G542X-CFTR or W1282X-CFTR, in combination with read-through agents and/or nonsense-mediated mRNA decay (NMD) inhibitors.^65^ Further experiments on HBE cells showed that **8** could also significantly enhance ENaC and TMEM16A channels’ activities. Such results suggest that CFTR amplifiers may alter the expression and/or function of other proteins involved in transepithelial ion transport,^65^ calling for the need for further investigations.

## 3 Targeting Heat Shock Proteins and Cochaperones

Two main chaperone systems are involved in the biosynthesis of the CFTR protein, mainly assisting the folding and assembly of the cytosolic domains of CFTR: the Hsp70/Hsc70 chaperone system is involved in early steps of CFTR folding,^66,67^ preferentially recognizing unfolded proteins, while the Hsp90 system is involved in later steps, binding to partially folded intermediates.^68^ These two complexes help CFTR to fold properly, protect the channel from aggregation, and trigger the degradation of non-native conformers.

### 3.1 Hsp70/Hsc70 Chaperone System

**Figure 3:**
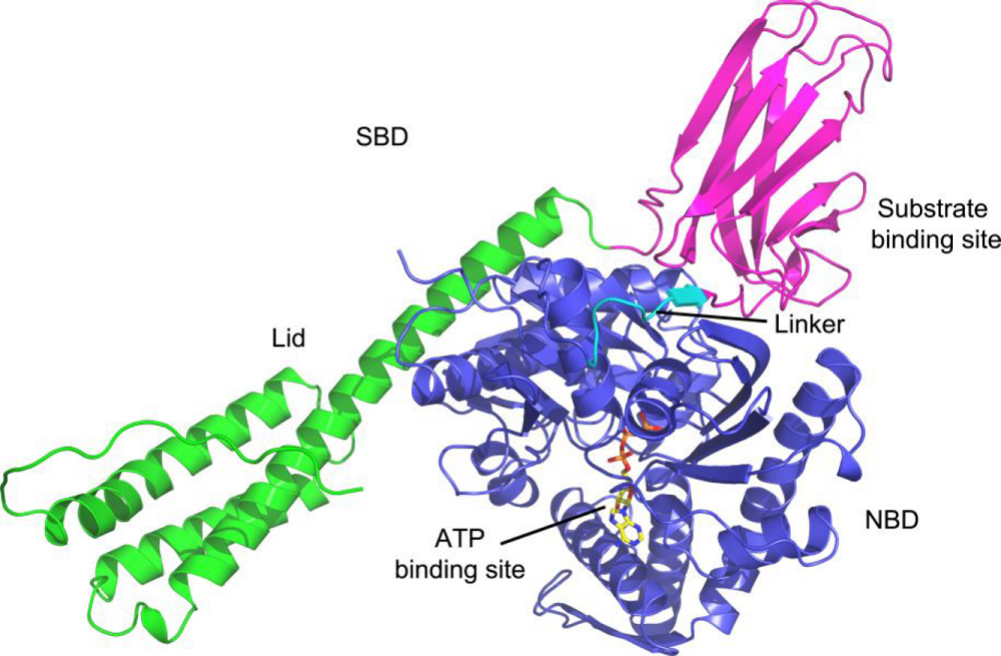
Predicted structure of Heat shock 70 kDa protein 1A by AlphaFold.^69^ The N-terminal nucleotide-binding domain (NBD) (also known as the ATPase domain) is represented in blue. The C-terminal substrate-binding domain (SBD) (magenta) contains a substrate-binding pocket that interacts with client/substrate proteins. An α-helical subdomain from the C-terminal side of SBD forms a flexible lid (green).

In the human cytosol, the major Hsp70 chaperones are the constitutively expressed heat shock cognate protein Hsc70 and its stress-inducible homologue Hsp70, which are closely related. Structure prediction using AlphaFold^69^ revealed that Hsp70 is formed by an amino-terminal ATP-binding domain (NBD), a C-terminal substrate-binding domain (SBD), and an α-helical subdomain that forms a flexible lid (Figure 3). Both Hsp70 and Hsc70 share an ATP-dependent mechanism that is regulated by two classes of cochaperones: DNAJs stimulate ATP hydrolysis and substrate binding to Hsp70/Hsc70 SBD, whereas NEFs promote the release of ADP and Hsp70/Hsc70 dissociation from the substrate.^70^ Proper folding of NBD1 of CFTR at the ER is highly dependent on Hsc70 and its cochaperone DNAJA1.^71^ Using a proteomic approach, it was found that more Hsc70 was associated with misfolded F508del-CFTR than with WT, consistent with the engagement of chaperones in trying to refold mutant CFTR.^72,73^ Direct evidence was obtained with DNAJA1 knockdown, which decreased CFTR folding and trafficking and increased the degradation of both WT- and F508del-CFTR.^74^ Instead, up-regulation of the inducible Hsp70 and its cochaperone Hsp40 was seen to lead to a modest but significant improvement in trafficking, stabilization, and activity of F508del-CFTR at the PM.^75,76^ However, the Hsc70/Hsp70 chaperone complex is also involved in CFTR degradation and in cell-surface quality control (QC). In the cytosol, Hsc70 in complex with the soluble E3 ubiquitin ligase CHIP binds more tightly to misfolded F508del-CFTR than to WT-CFTR, and thus the mutant protein is incapable of exiting the ER and is degraded by the proteasome.^73,77^ In parallel at the ER level, Hsc70 promotes the degradation of F508del-CFTR dependent on the membrane-anchored E3 ligases gp78 and RMA1.^74^ The Hsc70-CHIP complex also functions in the clearance system at the PM by promoting internalization by endocytosis and lysosomal degradation of misfolded CFTR.^77,78^ How these opposite roles of Hsc70 are balanced remains unclear.

Even if evidence suggests that targeting the Hsc/Hsp70 complex might have pleiotropic effects, it is believed that both profolding and antidegradation strategies could be therapeutically interestesting. For that, small molecules that target components of the Hsc/Hsp70 system can either block mutant CFTR degradation or promote its folding and therefore have potential applications as CF therapeutics.

**Figure 4:**
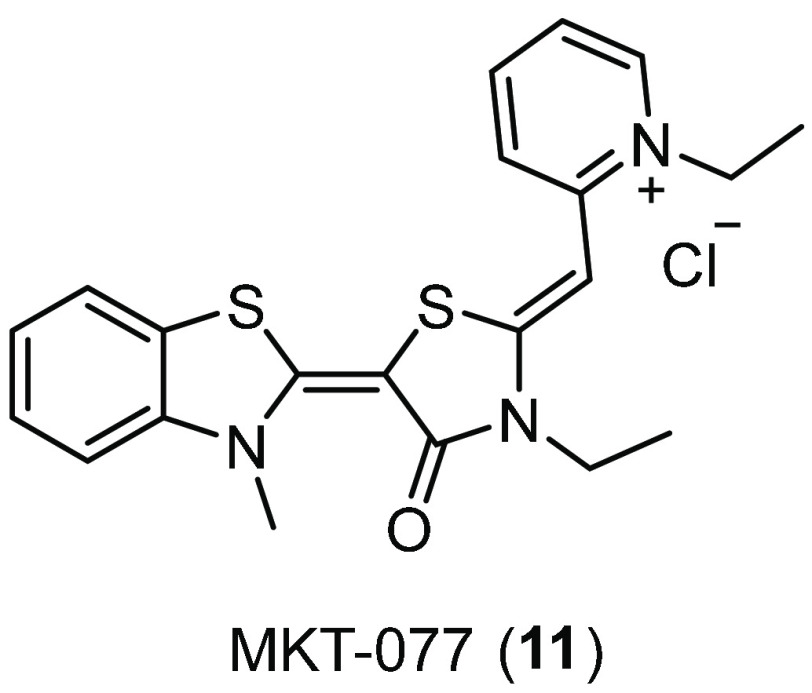
Structure of the allosteric Hsc70 inhibitor MKT-077 (**11**).

In recent years, small molecule inhibitors of Hsc70 have become available, and many are being studied for their antitumor properties. The growing evidence that Hsc70 inhibition can rescue defective cellular processing of mutant CFTR prompted the evaluation of these inhibitors’ activity on membrane trafficking of F508del-CFTR. For example, the rhodacyanine derivative MKT-077 (**11**, Figure 4) is an allosteric inhibitor with high affinity for the ADP-bound state of Hsp70.^79,80^ Young and co-workers proved that **11** could enhance levels of both mature protein and F508del-CFTR, by slowing turnover and allowing delayed maturation, respectively.^81^ Thus, **11** appears to increase the stability of F508del-CFTR against ER-associated degradation (ERAD), allowing the accumulation of further rescued protein. Furthermore, when combined with the corrector **2**, **11** was able to efficiently correct the trafficking defect of F508del-CFTR and boost the mutant channel activity.^81^ However, compound **11** was withdrawn from phase 1 clinical study due to its poor metabolic stability.^82^ Since then, many researchers have been looking for analogues of **11** with a similar effect on Hsc70 and improved safety and pharmacokinetic profiles.^83,84^ Despite that, an analysis of the efficacy of those new Hsc70 inhibitors on CFTR rescue is still missing, and we believe that it could be of great interest for the CF community.

**Figure 5:**
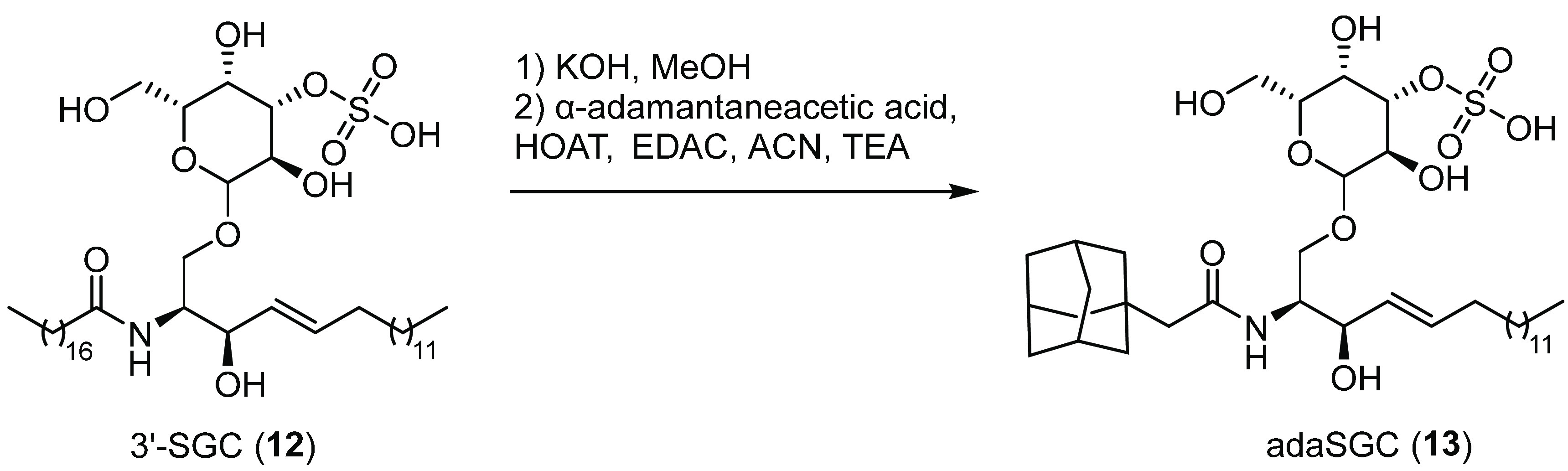
Chemical modification of 3′-SGC (**12**) to obtain the water-soluble analogue adaSGC (**13**), a Hsp40–Hsp70 chaperone complex inhibitor.^91^

Sulfogalactolipids (SGL), such as sulfogalactosylceramide (SGC), were found binding to a putative sulfatide binding site on a variety of Hsp70s.^85−88^ This sulfatide SGL binding site lies within the N-terminal ATPase domain of Hsp70,^85^ and SGL binding is known to decrease ATPase activity.^89^ On the basis of this knowledge, Whetstone and Lingwood synthesized a water-soluble analogue of SGC that could mimic the structural and functional features of the natural glycolipid.^90^ For that, commercially available 3′-sulfogalactosyl ceramide (3′-SGC, **12**, Figure 5) was subjected to deacylation, and the generated amine coupled to the carboxyl group of α-adamantaneacetic acid, yielding compound adaSGC (**13**, Figure 5) with an α-adamantane rigid frame.^90^ Single-turnover assays indicated that the resulting conjugate **13** inhibited the Hsp40-stimulated ATPase activity of the Hsp70 chaperone, with a *K*_i_ of ∼10 μM. Interestingly, **13** was seen to be associated with the N-terminal domain of Hsp70, directly hindering the Hsp40 binding site and reducing the C-terminal peptide binding. In transfected baby hamster kidney (BHK) cells, **13** increased the levels of immature F508del-CFTR, suggesting that inhibition of Hsp70s ATPase activity by **13** might suppress the ERAD pathway. Increased maturation and iodide influx, however, were observed only after low-temperature glycerol rescue of F508del-CFTR in **13**-treated cells.^91^ Furthermore, the binding site of these glycolipids is only loosely defined, and their selectivity has yet to be firmly established. Despite these insights, the authors did not investigate further the role of **13** on CFTR rescue but instead moved forward and proposed **13** as a new tool to manipulate mammalian glycosphingolipid metabolism in lysosomal storage disease.^92^

**Figure 6:**
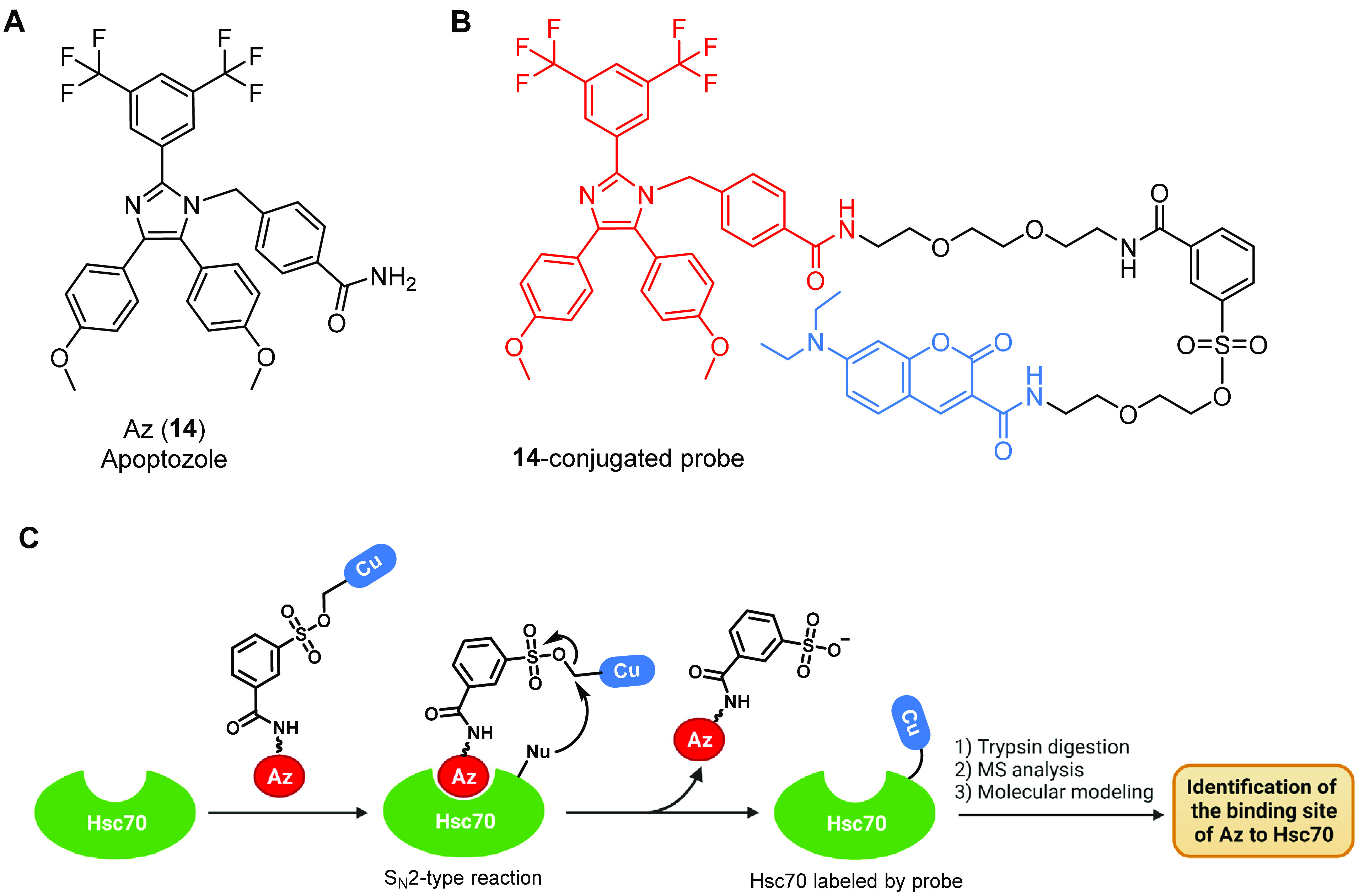
Structure of the Hsc70 ATPase activity inhibitor Az (**14**) in the study by Shin and co-workers and ligand-directed protein labeling technique used to determine the detailed binding mode of **14** to Hsc70. (A) Chemical structure of **14**. (B) Chemical structure of the probe employed in the ligand-directed protein labeling assay, where **14** (in red) is conjugated with diethylaminocoumarin (in blue) via a sulfonate group. (C) Schematic illustration of the strategy used to identify the binding site of **14** on Hsc70 using the **14**-conjugated probe. Cu = diethylaminocoumarin; Nu = a nucleophilic amino acid residue.^95^ Created with BioRender.com.

Looking for molecules with apoptosis-inducing activity as potential cancer therapeutics, Shin and co-workers used a cell-based assay to screen a previously synthesized imidazole library of 216 derivatives.^93,94^ One of them, Apoptozole (Az, **14**, Figure 6A), was seen to regulate apoptosis by inhibiting the function of Hsp70 and/or Hsc70.^94^ Further studies showed that **14** blocked the ATPase activity of Hsp70 by 55% at 200 μM, based on the malachite green assay, by binding to its ATPase domain, as demonstrated by affinity chromatography. To explore the utility of the Hsc70 inhibitor **14** in rescuing defective F508del-CFTR cellular processing, the same authors initially determined the detailed binding mode of **14** to Hsc70.^95^ For that, they employed a ligand-directed protein labeling method with a **14**-conjugated probe, obtained by connecting **14** to diethylaminocoumarin via a sulfonate reactive group (Figure 6B). Briefly, when the purified Hsc70 was incubated with the **14** probe, the binding of the **14** moiety promoted a S_N_2-type reaction of the sulfonate group with a nucleophilic amino acid residue located near the binding site of the protein (Figure 6C). This chemical event promoted protein labeling because the **14**-containing moiety was released from the probe. Then the labeled amino acids were identified by mass spectrometry (MS) after trypsin digestion. Next, molecular modeling studies of the complex between the **14** probe and Hsc70 were performed, suggesting that **14** might inhibit Hsc70 activity by interacting with the ATP binding pocket. Treatment of F508del-CFTR cells with nanomolar concentrations of **14** induced cAMP-stimulated CFTR chloride channel activity by increasing the expression of the mutant channel at the PM. Further measurements of the half-life of rescued F508del-CFTR suggested that **14** could also increase the stability of the cell-surface mutant CFTR. In addition, the study suggested that **14**-induced membrane trafficking of F508del-CFTR might be caused by the disruption of mutant CFTR association with Hsc70 and CHIP, thus suppressing its ubiquitination and causing escape from the ERQC.^95^ However, the authors did not investigate the effect of **14** on other CFTR processing events, and after this initial interest for **14** in CF, the group moved to study this small molecule for the development of new anticancer therapies.^96^

**Figure 7:**
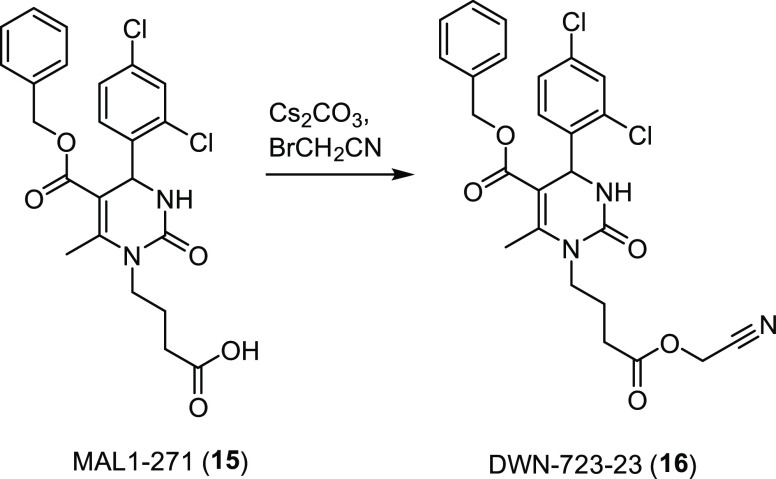
Chemical structure of the Hsp70 agonist MAL1-271 (**15**) and synthesis of DWN-723-23 (**16**), the most promising new derivative.^98^

In contrast to the inhibition of Hsc70, which has proven to be more effective in preventing the growth of a variety of cancer cells, researchers have been prompted to look for small molecules that could cause an increase of the activity of Hsp70 or other chaperones that might prevent protein aggregate accumulation. Indeed, enhanced Hsp70 activity by compounds that act as Hsp70 agonists could improve CFTR folding and prove beneficial to promote F508del-CFTR rescue. With this idea, Brodsky and co-workers monitored the stability of F508del-CFTR in the presence of MAL1-271 (**15**, Figure 7), a previously reported dihydropyrimidinone activator of Hsp70. In vitro studies showed that **15** accelerated the ATPase and protein-folding activity of Hsp70 in the presence of Hsp40 by binding to the cochaperone binding site of Hsp70 and thus regulating Hsp70–Hsp40 complex assembly.^97^ Using an immunoblot assay, they examined the effect of **15** on the ER glycosylated and Golgi glycosylated forms of F508del-CFTR. They found that 30 μM of **15** increased the amount of the ER glycosylated form (∼2.1-fold) to a greater extent than the corrector **2** without increasing the level of the Golgi glycosylated form of CFTR. Perhaps this reflects the fact that a portion of the immature protein had folded into a proteasome-resistant state. Based on these results, they assayed 12 derivatives of **15**, which were synthesized through modification of the free acid at the end of the flexible hydrocarbon chain. Interestingly, compound DWN-723-23 (**16**, Figure 7), generated by alkylation of the cesium salt of **15** to obtain a derivative containing a nitrile instead of the free acid, was almost equally active as **15** in the F508del-CFTR maturation assay. Therefore, although further modifications are in order to increase the activity of this class of derivatives, the authors speculated that the cyanomethyl ester moiety of **16** could convey biological absorption potential superior to that of the carboxylate in **15**. Meanwhile, this function should maintain similar binding affinity due to the polar nitrile group that might act as hydrogen bond acceptor. Finally, none of the derivatives exhibited cellular toxicity nor induced cellular stress response pathways, in contrast to what was observed with Hsp70 inhibitors, which are mostly cytotoxic. Taken together, these results serve as a gateway for the development of new Hsp70 agonists and for further optimization of the pharmacokinetic properties of this pyrimidinone–peptoid class of compounds.^98^

### 3.2 Hsp90–Aha1 Chaperone Complex

The Hsp90 cytosolic chaperone system has been broadly implicated in the folding process of more than 300 specific client proteins,^99^ including nascent CFTRs.^100,101^ Initially, the proof-of-concept of the role of Hsp90 in CFTR post-translational folding was demonstrated with Hsp90 inhibitors such as geldanamycin, a 1,4-benzoquinone ansamycin antibiotic.^102^ The immature CFTR molecule was detected in association with Hsp90, and this interaction was found to have a major impact on the fate of nascent CFTR.^100^ Therefore, geldanamycin, binding to the ADP/ATP-binding pocket of Hsp90, was able to nearly completely abrogate the maturation of nascent WT-CFTR and enhance its degradation. These results provided the evidence of the role of Hsp90 in the maturation of newly synthesized, incompletely folded, or assembled cytoplasmic CFTRs.^100^

**Figure 8:**
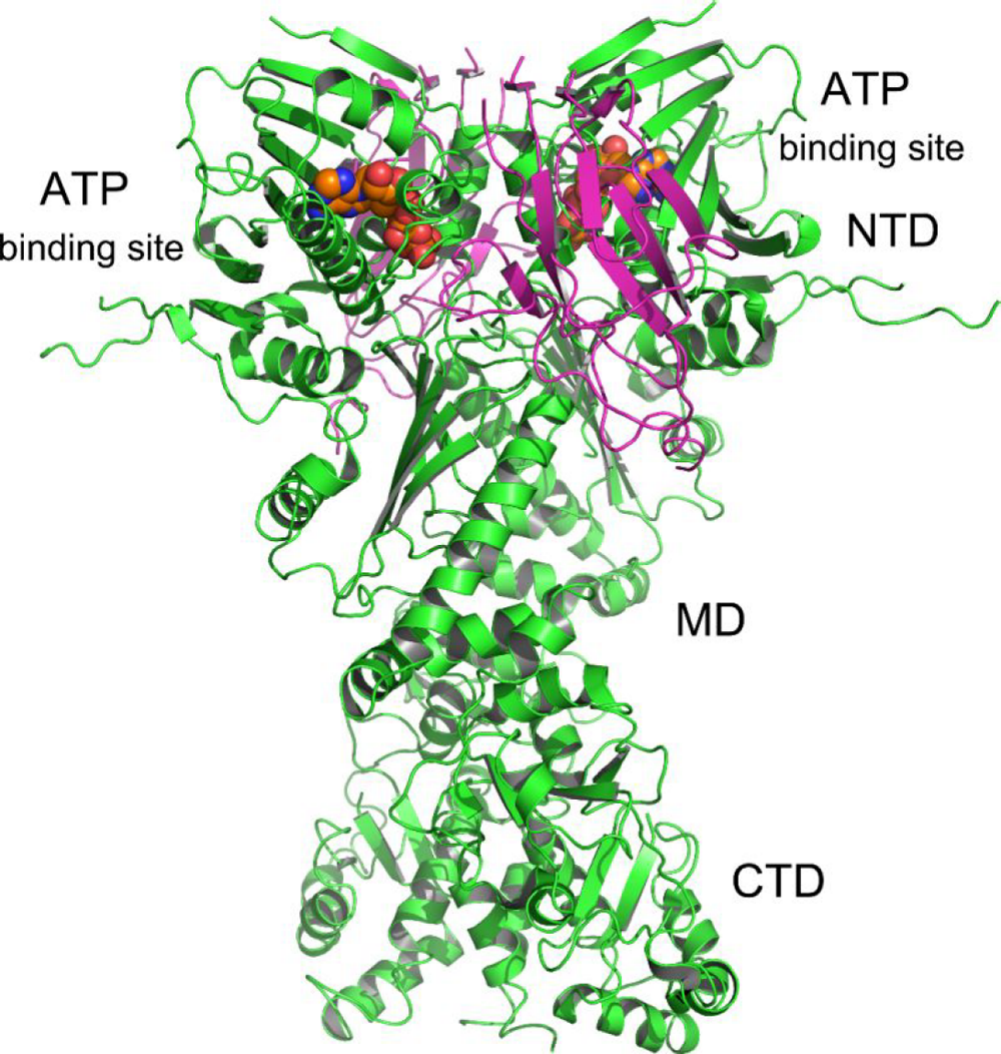
Crystal structure of full-length yeast Hsp90 (green) in complex with an ATP analogue (sphere, orange) and the cochaperone p23/Sba1 (magenta) (PDB: 2CG9).^109^

Hsp90 is a flexible homodimer consisting of three domains per monomer: the N-terminal domain (NTD) containing the ATP-binding site, the middle domain (MD) where the unfolded client proteins are assembled, and the C-terminal domain (CTD) (Figure 8).^68^ The function of Hsp90 is regulated by ATP-induced large conformational changes, which represent the rate-limiting step of the formation of the Hsp90 catalytically active state.^103^ Among the variety of cochaperones that participate to the ATPase cycle of Hsp90,^104,105^ the activator of the Hsp90 ATPase (Aha1) had a major role.^106^ By MS approaches, Balch and co-workers demonstrated that the C-terminal domain of Aha1 binds to the NTD of Hsp90.^101^ This interaction accelerates the formation of an N-terminally closed state of Hsp90 that triggers the assembling of the unfolded substrates on Hsp90 MD (Figure 8).^101,107^ Subsequently, the hydrolysis of ATP leads to the opening of the NTD of Hsp90 dimer and to the release of the mature client protein.^108^

**Figure 9:**
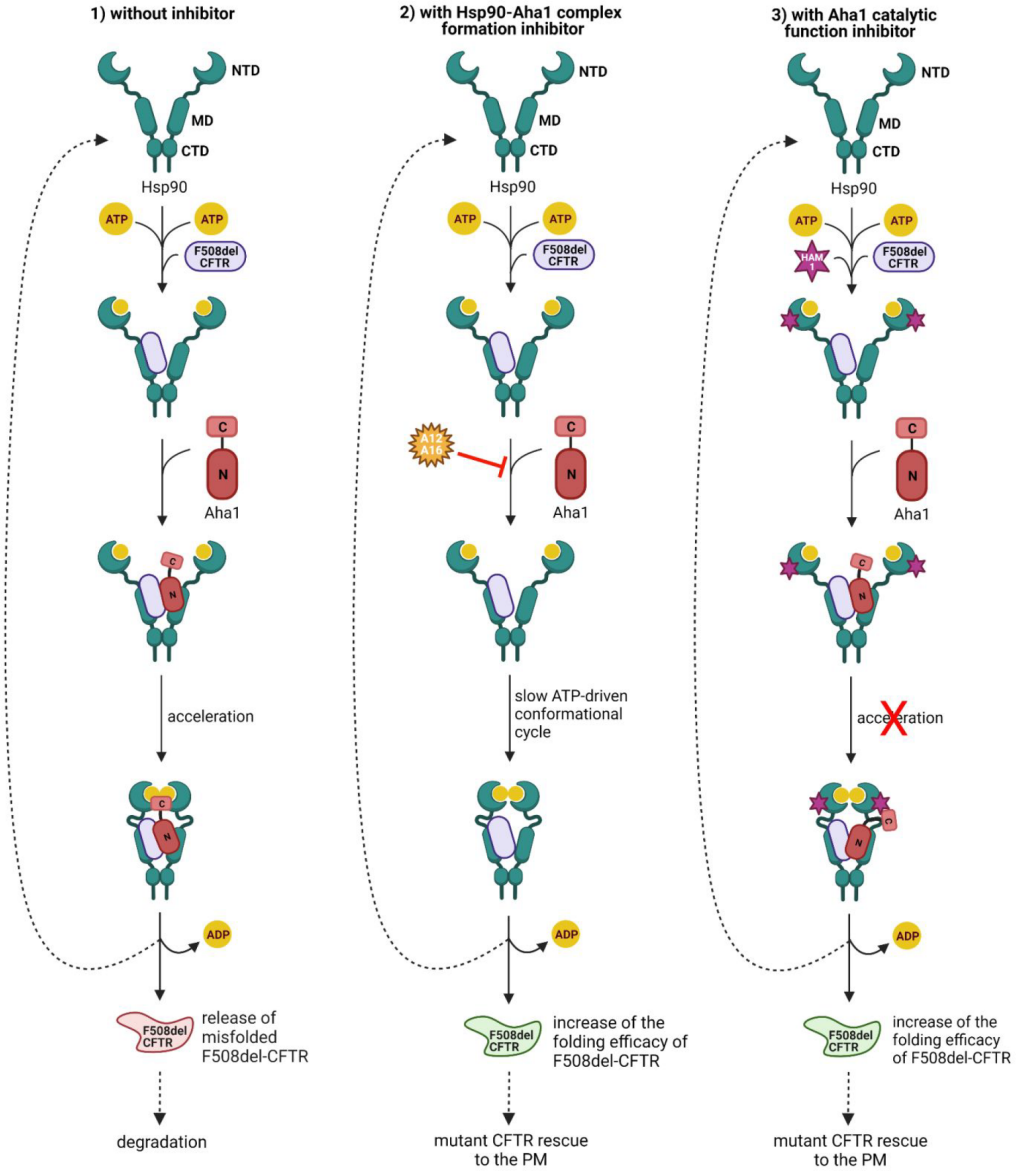
Hsp90 ATPase chaperone cycle and potential CF therapeutic strategies. (1) The client protein, such as F508del-CFTR, is loaded onto the middle domain (MD) of Hsp90. Aha1 increases Hsp90 ATPase activity, thus contributing to the closing of the N-terminal domain (NTD). Finally, ATP hydrolysis causes the release of the misfolded CFTR channel. The open conformation of Hsp90 is restored for the next chaperone cycle. F508del-CFTR is targeted by the QC system and degraded. (2) A12 (**17**) and A16 (**18**) might inhibit Hsp90–Aha1 chaperone complex formation, slowing down the conformational changes of Hsp90 and enhancing its folding efficacy. (3) HAM-1 (**19**) might interfere with the catalytic function of the cochaperone Aha1 C-terminal domain (C), without preventing Aha1 N-domain (N) binding to Hsp90 MD. This inhibition might increase the dwell time with F508del-CFTR and therefore enhance its folding efficacy. Created with BioRender.com.

When F508del-CFTR is processed by the Hsp90–Aha1 complex, the mutation impairs folding and kinetically restricts F508del-CFTR to a folding intermediate, which is prematurely degraded by the ERAD pathway (Figure 9, panel 1).^56,101^ Strikingly, the knockdown of Aha1 was seen rescuing the trafficking of F508del-CFTR to the cell surface and restoring channel function.^56^ This suggests that the Hsp90–Aha1 machinery governing the folding of F508del-CFTR can be manipulated pharmacologically to promote folding and transport of the mutant channel to the PM. Accordingly, disrupting the interaction of Hsp90 with Aha1 may slow down the ATP-driven conformational cycle of Hsp90, increasing the folding efficacy of F508del-CFTR and protecting the mutant fold from degradation.^101^

**Figure 10:**
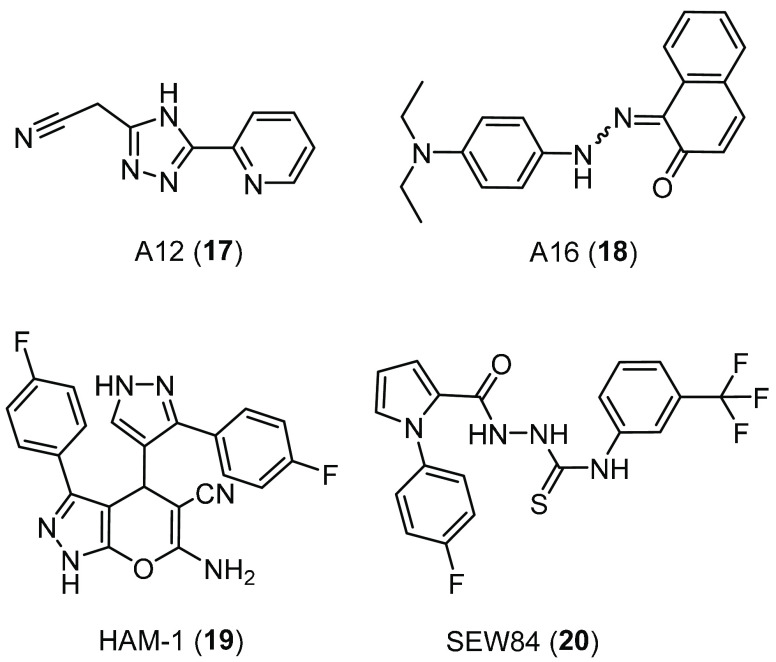
Chemical structures of the Hsp90–Aha1 complex inhibitors A12 (**17**), A16 (**18**), and of the Aha1-stimulated Hsp90 ATPase activity inhibitors HAM-1 (**19**) and SEW84 (**20**).

The Hsp90–Aha1 complex formation can be, in principle, inhibited with protein–protein interaction (PPI) inhibitors, as shown by Obermann and co-workers (Figure 9, panel 2).^110^ For that, they adapted the Hsp90–Aha1 complex to serve as target for inhibitor screening using amplified luminescence proximity homogeneous assay (Alpha Technology). Using this system, 14,400 drug-like molecules of the Maybridge HitFinder collection were tested, and among them, eight candidates inhibited Hsp90–Aha1 interaction. Next, the drug-like molecules were assayed for their potency to preserve the CFTR residual channel activity in BHK cells stably expressing F508del-CFTR, using the iodide efflux assay. The 1,2,4-triazolic compound A12 (**17**, Figure 10) and the naphthalenone A16 (**18**, Figure 10) increased the iodide efflux ∼2.5-fold compared to that of the untreated control. Further biological evaluations showed that **17** and **18** were most effective in combination with the corrector **2**, increasing the iodide transport ∼25-fold or ∼15-fold, respectively, compared to untreated cells. Thus, the authors suggested that the two molecules, by acting as Hsp90–Aha1 chaperone complex formation inhibitors, could be beneficial to further enhance F508del-CFTR channel activity as they showed potentiated synergistic effects in combination with **2**.^110^ However, the regulation of the Hsp90–Aha1 interaction through PPI inhibitors is still going through an embryonic stage and requires further research. Furthermore, **17** inhibited multiple targets, including (1,6)-β-glucan synthesis^111^ and Notum carboxylesterase activity, which recently led to its application in the treatment of central nervous system disorders and in cancer and metabolic disorder therapy.^112^ For these reasons, **17** is unlikely to represent a CF drug scaffold.

In a different proof-of-principle study, Buchner and co-workers showed that a drug-like small molecule could specifically interfere with the catalytic function of the cochaperone Aha1, without affecting complex formation with Hsp90 (Figure 9, panel 3).^113^ For that, they screened a library of ∼15,000 chemical compounds from ChemDiv by a FRET-based assay that monitored the interaction between Hsp90 and Aha1 and identified nearly 40 compounds as possible modulators. Among them, the dihydropyranopyrazole HAM-1 (**19**, Figure 10) was the strongest inhibitor, able to almost completely suppress the stimulatory effect of Aha1 on the Hsp90 ATPase without preventing binding of Aha1 to Hsp90, as determined by SPR measurements. NMR studies revealed that **19** could bind to the Hsp90 NTD, thus sterically blocking its interaction with Aha1that is required to accelerate the formation of the Hsp90 N-terminally closed state. Instead, the Hsp90 MD appeared to be unaffected in its interaction with Aha1 N-domain by **19**, and therefore, this inhibitory compound could not dissociate the Aha1–Hsp90 complex. Further in vivo studies demonstrated that **19** affected the activation and processing of Hsp90–Aha1-dependent client proteins. Then, because Hsp90–Aha1 machinery targets F508del-CFTR for degradation, the effect of **19** on this misfolded protein stability was evaluated. As expected, in the presence of **19**, higher amounts and a prolonged lifetime of F508del-CFTR were observed.^113^

In 2020, Singh et al. identified a novel inhibitor of the Aha1-stimulated Hsp90 ATPase activity, the isothiosemicarbazide SEW84 (**20**, Figure 10), using a modified quinaldine red-based HTS.^114^**20** emerged to bind to the C-terminal domain of Aha1, causing the weakening of its binding to Hsp90 without affecting the basal ATPase activity of Hsp90. Therefore, similarly to **19**, **20** could maintain the basal activity of Hsp90, potentially avoiding the toxic effects reported with the use of common Hsp90 inhibitors.^114−116^ However, the authors only evaluated the effects of **20** on tau phosphorylation and on the trophic activity of androgen receptor variants but did not investigate its impact on CFTR protein folding.^114^ Nevertheless, these results highlight the potential benefits of small molecule inhibitors of the Aha1-stimulated Hsp90 ATPase activity in managing proteostatic diseases such as CF, and hopefully, this will be further substantiated with in vivo experiments.

## 4 Targeting Ubiquitination/Deubiquitination Enzymes

The network of the ERQC factors, known as ERAD, is responsible for disposing of newly synthesized F508del-CFTRs that fail to reach their proper conformations.^117^ Misfolded F508del-CFTR accumulates in a kinetically trapped conformation, which is retained in the ER and ubiquitinylated by the sequential action of a ubiquitin activating enzyme (E1), a ubiquitin-conjugating enzyme (E2), and a ubiquitin ligase (E3). Next, the poly-ubiquitinylated F508del-CFTR is prematurely degraded by the UPS, leading to reduced PM expression of the mutant channel.^118^ The ubiquitination process is regulated by deubiquitinating enzymes (DUBs) that catalyze the deconjugation of ubiquitin chains from substrates. At the peripheral level, ubiquitination and deubiquitination processes regulate endocytosis, endocytic recycling, and lysosomal degradation of CFTRs.^119,120^ Therefore, the plasma abundance of this membrane protein is largely dependent on the balance between ubiquitin ligation by E3 ligases and deubiquitination by DUBs.

Efforts to manipulate the UPS to promote the functional expression of the CFTR channel have focused on different strategies, such as (i) inhibiting the proteasome, (ii) inhibiting E1 or E3 ligases, and (iii) activating endogenous DUBs. However, all three approaches have several limitations, due to their widespread role in proteostasis that may lead to off-target effects. Early studies investigated whether proteasome inhibitors could rescue F508del-CFTR, even though it is a relatively nonspecific way to target ubiquitin-dependent protein degradation.^121^ However, it was shown that proteasome inhibition leads to accumulation of insoluble, multi-ubiquitinylated F508del-CFTR proteins, with no detectable increase in the level of folded CFTRs.^122^ Based on these results, targeting earlier and more specific steps in the ERAD cascade, such as enzymes involved in the ubiquitination or deubiquitination process, might be more reasonable to achieve an increase of the pool of correctable F508del-CFTR.

### 4.1 Ubiquitin Ligase RNF5/RMA1

In 2006, Younger and co-workers identified an ER-associated ubiquitin ligase complex, containing the membrane-anchored E3 ubiquitin ligase RNF5 (also known as RMA1), the E2 Ubc6e, and the transmembrane QC factor Derlin-1.^123^ This complex cooperates with the cytosolic Hsc/Hsp70-CHIP E3 ligase complex to monitor the conformation and to triage nascent WT-CFTR and F508del-CFTR.^124^ However, while CHIP QC checkpoint inspects the folding status of CFTR’s cytosolic domains,^71,77,124^ the RMA1 QC checkpoint senses the assembly status of amino terminal regions of CFTR.^123^ Moreover, the CHIP E3 complex performs its function after CFTR’s NBD2 synthesis (post-translational role), while RNF5 E3 complex acts prior to NBD2 synthesis (co-translational role). Although these two sequential QC checkpoints are both responsible for F508del-CFTR degradation, RNF5 was seen to be particularly relevant, as its loss by RNA interference strongly increases the folding of F508del-CFTR and synergizes with the bithiazole corrector **5** (Figure 1)^125^ to rescue F508del-CFTR folding.^126^ Given our group’s interest in the identification of novel targets and chemical compounds for the development of innovative therapeutic approaches for CF, we screened a siRNA library targeting known CFTR interactors.^127^ Our analysis showed that silencing RNF5 elicited a 70–80% increase in F508del-CFTR function in the microfluorimetric assay based on the halide-sensitive yellow fluorescent protein (HS-YFP) and displayed an additive effect with the known corrector **2**. As validation of in vivo efficacy of RNF5 modulation, RNF5 knockdown in F508del-CFTR transgenic mice exhibited improved intestinal absorption and increased CFTR activity in intestinal epithelial cells, relative to animals expressing WT-RNF5.^127^ These findings validated RNF5 as a drug target for CF and provided the basis for the development of RNF5-targeting molecules that could inhibit its activity.

**Figure 11:**
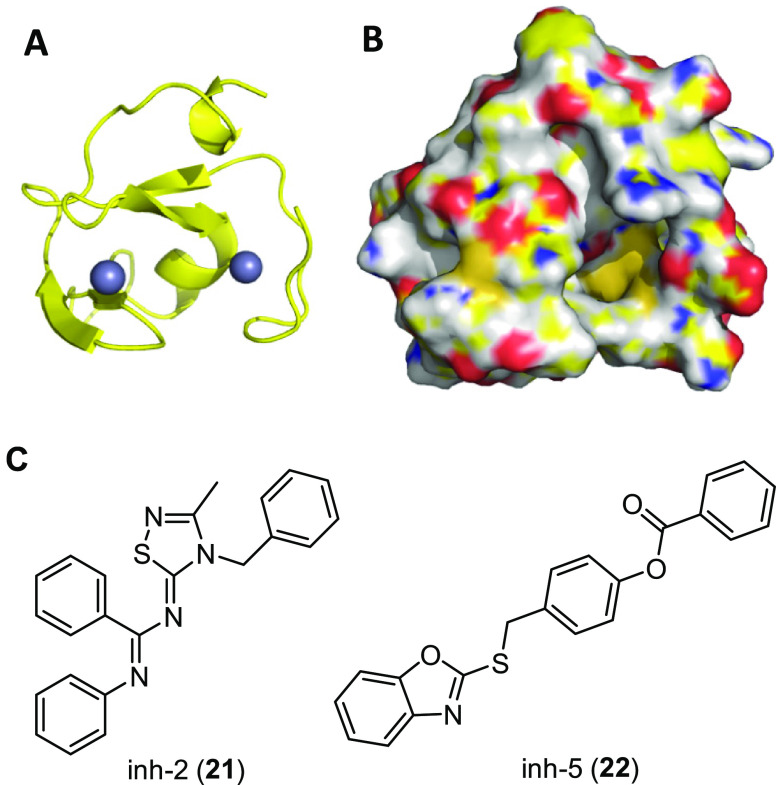
Representative conformational state of the RNF5 ring domain extracted from the molecular dynamics trajectory conducted on the model built by homology modeling that was used to select RNF5 ligands.^128^ (A) Cartoon representation of RNF5 ring domain; zinc ions are represented as spheres. (B) Surface representation of RNF5 ring domain. (C) Chemical structures of hits inh-2 (**21**) and inh-5 (**22**) identified from the primary screening.

With this aim, using a computational approach, we generated a homology model of RNF5 RING domain (Figure 11A,B) and performed high-throughput docking selecting a first set of 1623 ligands.^128^ A second diversity set of 1000 ligands, based on molecular fingerprinting chemical diversity, was extracted from the LifeChemicals database. In total, 2623 molecules were purchased and tested as F508del-CFTR correctors, using the HS-YFP functional assay in a bronchial epithelial cell line (CFBE41o-). This primary biological screening identified two potential hit compounds having a clear dose-dependent effect, the thiadiazolylidene derivative inh-2 (**21**, Figure 11C) and the benzooxazolylthiolic compound inh-5 (**22**, Figure 11C). To evaluate the best hit as a F508del-CFTR corrector, we used electrophysiological techniques on human primary bronchial epithelia. As a result, **21** increased the F508del-CFTR-mediated current, whereas **22** had no activity as a corrector. The lack of consistency between results obtained with **22** in immortalized and primary bronchial cells could be explained by assuming a possible effect of **22** on other targets than RNF5 that, in primary cells, hinder mutant CFTR rescue. On the other hand, ubiquitination experiments confirmed that **21** decreased ubiquitination of mutant CFTR, thus stabilizing its mature form. To further investigate the mechanism of action of the putative RNF5 inhibitor **21**, we exploited known RNF5 downstream targets, such as the regulator of basal autophagy ATG4B and the actin cytoskeleton factor Paxillin. Functional and biochemical experiments confirmed that **21** decreases the ubiquitinylated form of ATG4B and increases the basal level of autophagy, while scratch/wound healing assays confirmed that **21** increases cell motility, consistent with what has been described for RNF5 knockdown. Taken together, all of these data demonstrated that **21** could act as RNF5 inhibitor able to rescue the F508del-CFTR trafficking defect and pioneered **21** optimization through a medicinal chemistry campaign, which is currently ongoing.

### 4.2 Ubiquitin-Activating Enzyme E1

**Figure 12:**
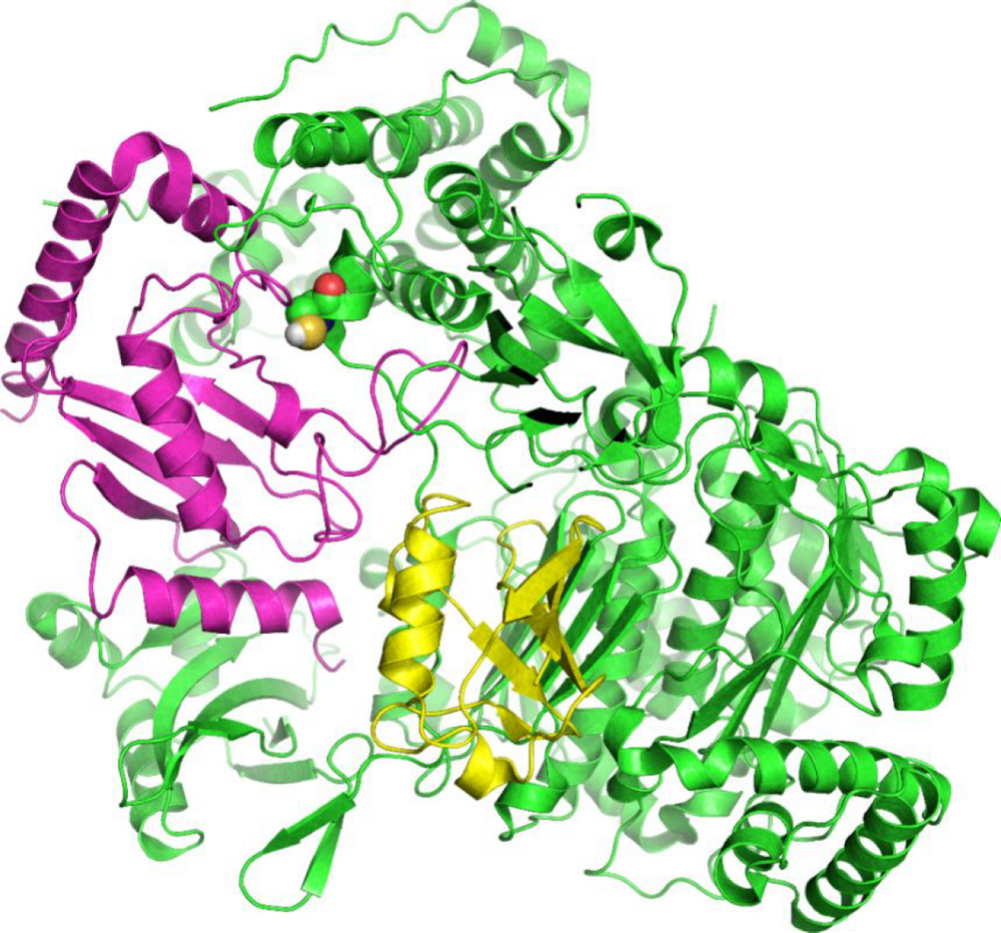
Complex of E1 (green), E2 (magenta), and ubiquitin (yellow) of *S. pombe* (PDB: 5KNL).^131^ Catalytic Cys593 of E1 is shown as spheres.

Another way to achieve F508del-CFTR stabilization through suppression of the ubiquitin-dependent degradation pathway may be the inhibition of the ubiquitin-activating enzyme E1. While E3s determine the substrate specificity of ubiquitination and, therefore, some hundreds of different mammalian E3s are involved in different cellular processes, there is just one major E1 in human.^129^ Whereas the proteasome represents the final destination for many ubiquitinylated proteins, E1 is the common first step in ubiquitination, thus activating and transferring ubiquitin (Ub) to tens of different E2s involved in different downstream pathways (Figure 12).^130^ Indeed, compounds developed to inhibit E1 enzyme could enable F508del-CFTR to pass through both sequential cytosolic and ER-associated QC checkpoints, thus efficiently suppressing F508del-CFTR degradation.

**Figure 13:**
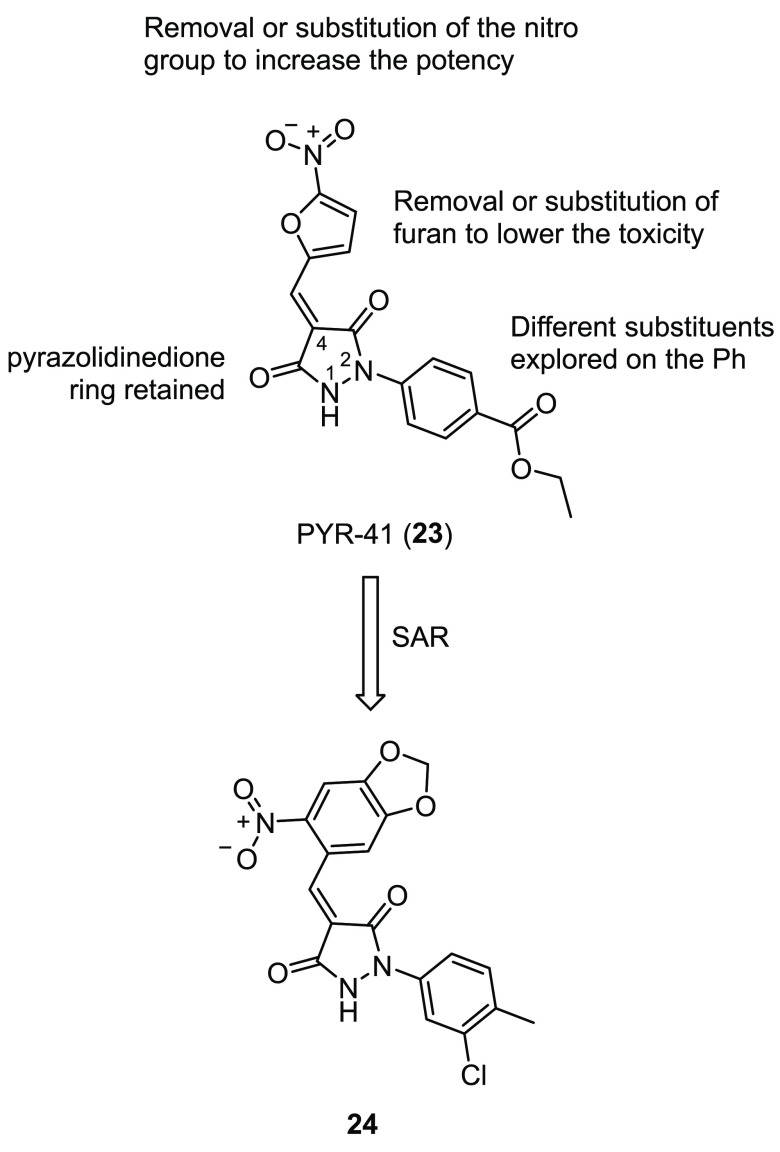
Depiction of the SAR strategy around E1 inhibitor PYR-41 (**23**) and structure of the optimized analogue **24**.^134^

Therefore, using the pyrazone PYR-41 (**23**, Figure 13), a previously reported E1 inhibitor used in cancer therapy,^132^ Brodsky and co-workers showed that it is possible to significantly increase F508del-CFTR stability, trafficking to, and activity at the PM when a E1 inhibitor is combined with different types of correctors.^133^ However, the use of **23** is limited due to its toxicity, perhaps linked to its 5-nitrofuroyl moiety. To identify small molecule analogues with lower toxicity and increased potency, the authors performed a SAR exploration around **23** by purchasing 22 different compounds from Sigma-Aldrich.^134^ All of the analogues left the pyrazolidinedione core unaltered while bearing different combinations of substituents in the 4-position of the central ring and on the phenyl ring in the 2-position. In particular, they focused on the removal of either nitro or furan moieties, while in some cases, the furan ring was replaced with different phenyl ring systems. In addition, some compounds had unique substituents at the 4-position of the pyrazolidinedione core, such as an indolinone, a pyrimidinetrione, or an isopropyl moiety. Other modifications included the methylation of the central pyrazolidine or the exploration of different substituents on the phenyl ring in the 2-position. These modifications are summarized in Figure 13. Ubiquitination experiments of both WT- and F508del-CFTR showed that the analogue **24** (Figure 13), bearing an electron-rich nitrobenzodioxolyl substituent in the 4-position and a 3-chloro-4-methylphenyl ring in the 2-position, markedly inhibited ubiquitination, meanwhile lacking the toxicity of the parent compound **23**. Further in vitro experiments showed that when corrector **2** was combined with **24**, a significant increase in maturation, expression, and activity of F508del-CFTR at the PM was observed in comparison to the combination of **23** and **2**. Finally, in silico modeling studies confirmed the proposed mechanism of action of **23** and **24**, with the molecules binding through extensive hydrogen bonds to a pocket near the active Cys593 of E1 (Figure 12). Overall, these results suggested that the suppression of F508del-CFTR ubiquitination by an E1 inhibitor could synergize with other known correctors, thus opening the way for further optimization of **24**.^134^ However, we should consider that **23** and **23**-related compounds were first proposed as therapeutics in cancer for their capacity to kill transformed cells.^132^ During the characterization of **23**, Yang and co-workers suggested that **23** could potentially function by covalently modifying E1, perhaps through heteroconjugate addition of the E1 cysteine residue to the α,β-unsaturated pyrazolidinedione.^132^ Subsequent studies on chemical reactivity of **23** provided some insight into the covalent protein cross-linking potential and partial target selectivity of **23**.^135^ Indeed, along with the inhibition of E1, **23** emerged to have equal or greater inhibitory activity against several DUBs.^135^ Furthermore, **23** mediated cross-linking of specific protein kinases such as Bcr-Abl and Jak2, causing the inhibition of their signaling activity.^135^ Therefore, further studies on **24**’s mechanism of action and selectivity are needed in order to address possible side effects and to refine the hit optimization strategy for this emerging class of E1 inhibitors.

### 4.3 Deubiquitinating Enzymes DUBs

The human genome encodes approximately 100 DUBs, among which some are responsible for the deubiquitination of WT-CFTR, although their role in the PM turnover of conformationally defective F508del-CFTR mostly remains unclear. Very recently, Colecraft and co-workers successfully demonstrated that selective ubiquitin chain removal could rescue trafficking-deficient CFTR via the development of engineered DUBs (enDUBs).^136^ For this proof-of-concept study, they prepared an enDUB comprising the catalytic component of ubiquitin-specific protease USP21 (enDUB-U21) and engineered CFTR protein to probe the impact of six distinct CF-causing mutations of class II and class IV that all impair channel surface density. Applying corrector **2** in combination with enDUB-U21 afforded a synergistic increase of four out of six of the CFTR mutants’ surface density from flow cytometry experiments. Strikingly, two mutations (N1303K and 4279insA) displayed expression levels equivalent to those of WT following the same treatment. Moreover, human embryonic kidney (HEK293) cells coexpressing enDUB-U21 and either N1303K CFTR or 4326ΔT CFTR yielded substantially increased **1**-potentiated currents. In order to enable enDUB-mediated functional rescue of endogenous CFTR channels, the authors adapted a selective nanobody (E3h) against CFTR to the enDUB-U21 system (enDUB-U21_CEE3h_). Remarkably, in Fischer rat thyroid (FRT) epithelial cells expressing the pharmacotherapy-resistant N1303K mutation, enDUB-U21_CEE3h_ in combination with the potentiator **1** and the corrector **2** rescued N1303K CFTR currents to ∼40% of WT levels. Furthermore, in the same cell system, enDUB-U21_CEE3h_ synergized with Trikafta to increase N1303K CFTR currents up to 80% of WT levels. Concerning the F508del mutation, the authors exploited the previously reported Cerulean-nanobody T2a^137^ in order to stabilize the F508del-CFTR channel upon binding of enDUB-U21_CET2a_. In FRT cells expressing F508del-CFTR, the enDUB-U21_CET2a_/**1**/**2** combination substantially rescued F508del-CFTR to ∼45% of WT levels. Notably, enDUB-U21_CET2a_ in combination with Trikafta increased F508del-CFTR currents to beyond WT levels.^136^ Although the application of enDUBs finds its place in gene therapy, the authors hypothesized that this therapeutic modality could be of inspiration for bivalent small molecules able to induce endogenous DUBs to target specific substrates, too.

**Figure 14:**
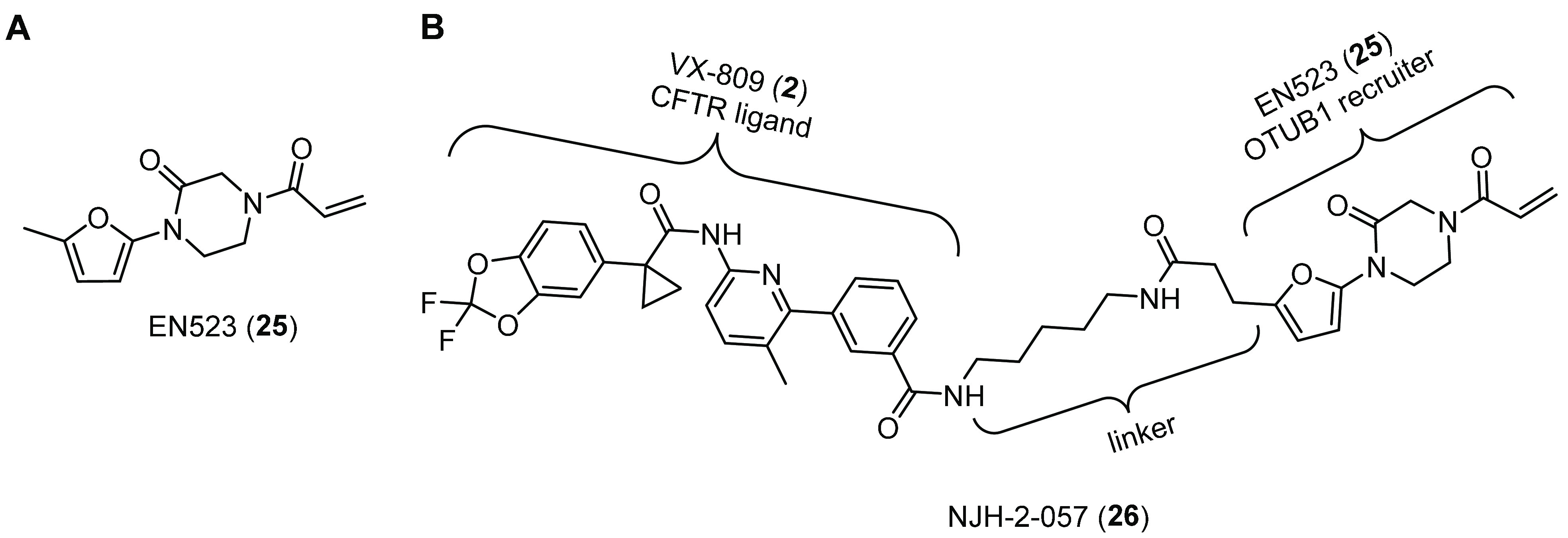
Deubiquitinase-targeting chimera (DUBTAC) for CFTR stabilization in study by Henning and co-workers.^141^ (A) Chemical structures of OTUB1 recruiter EN523 (**25**). (B) DUBTAC NJH-2-057 (**26**) synthesized by linking the CFTR ligand VX-809 (**2**) to the OTUB1 recruiter **25** through a C5 linker.

In both academic and industrial settings, targeted protein degradation (TPD) and targeted protein stabilization (TPS) have recently emerged as powerful drug discovery approaches.^138−140^ Proteolysis targeting chimeras (PROTACs) are the earliest example of TPD effectors and consist of heterobifunctional molecules formed by a protein-targeting ligand linked to an E3 ligase recruiter. Their purpose is to achieve the proteasomal degradation of a specific protein through induced proximity of the substrate with the E3 ubiquitination enzyme.^138−140^ Recently, Henning and co-workers extended induced proximity paradigm to develop a heterobifunctional stabilizer, such as a deubiquitinase-targeting chimera (DUBTAC) for CFTR stabilization.^141^ Using chemoproteomic approaches, the authors identified OTUB1 as a candidate DUB for covalent ligand screening and the acrylamide EN523 (**25**, Figure 14A) as a OTUB1 recruiter. Further evaluations showed that the cysteine-reactive acrylamide of **25** was able to selectively target the noncatalytic cysteine C23 of OTUB1, without interfering with its deubiquitination activity. Then the authors synthesized two different DUBTACs by linking **25** to the corrector **2** through C3 or C5 alkyl linkers. Between them, treatment of CFBE41o- cells expressing F508del-CFTR with the C5-DUBTAC NJH-2-057 (**26**, Figure 14B) showed a dose–responsive increase of CFTR stabilization, based on Western blotting data. Proteomic analysis of treated cells confirmed that CFTR was among the most stabilized proteins and showed that CFTR stabilization occurred through ∼60% of OUTB1 occupancy. These data suggested that F508del-CFTR stabilization by a fully synthetic DUBTAC might be possible, and the relatively minimal OTUB1 occupancy of the covalent **26** might still allow endogenous DUB function.^141^ However, no demonstration of increased CFTR mutants’ surface density and activity by **26** was obtained from this proof-of-concept study. Furthermore, the alteration of deubiquitination process is likely to lead to dysregulation of other biological networks DUB-associated. Indeed, OTUB1 has recently emerged as an essential regulator of a variety of physiological processes, such as immune signaling and DNA damage response, although its functions remain largely unclear.^142^ For that, elucidation of the mechanism of deubiquitination of CFTR and better understanding the consequences on protein homeostasis are pivotal prior to consider DUBTACs or enDUBs as CF therapeutic strategies.

## 5 Targeting Poly-ADP Ribose Polymerases

**Figure 15:**
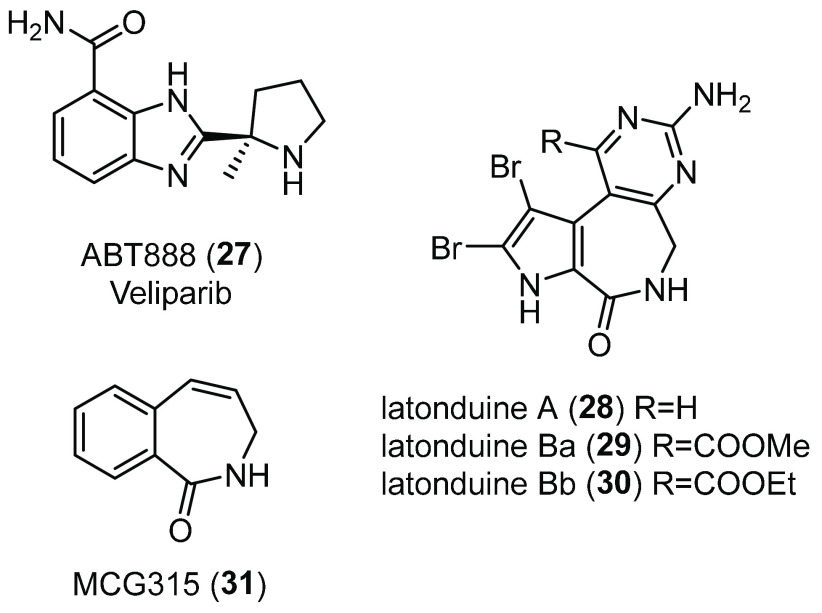
Chemical structures of the PARP-1 inhibitor ABT888 (**27**),^152^ the alkaloids latonduines (**28**–**30**), and MCG315 (**31**), a more potent derivative of **28**.^153^

The family of nuclear enzymes poly(ADP ribose) polymerases (PARPs) is a group of 17 enzymes that catalyze the attachment of polymers of ADP ribose to different target proteins, a modification known as poly-ADP (ribosyl)ation (PARylation).^143,144^ The PARP family has been involved in the regulation of multiple basal cellular processes, including DNA repair, cell division, protein homeostasis, oxidative stress, and viral infection. PARP-1–6 have shown to transfer polymeric chains of ADP ribose to substrates inside the cell, whereas PARP-7–17 are either presumed or proven to attach one ADP ribose unit per time.^145−147^ PARP-1 is the most abundant and best-characterized isoform of the PARP family for its involvement in DNA damage repair and genome maintenance.^146^ Consequently, PARP-1 has been primarily considered to be an attractive drug target, and several inhibitors are currently under investigation for cancer therapy.^148^ In CF, the presence of defective CFTR appears to produce a redox imbalance in epithelial cells and extracellular fluids and to cause an abnormal generation of reactive oxygen species (ROS).^149^ Thus, CF patients display increased susceptibility to oxidative-induced DNA damage, although this appears to be independent of clinical status.^150^ In view of the central role of PARP-1 in cellular stress response,^143^ Thomas and co-workers decided to investigate its role in CF and demonstrated that PARP-1 activity is 2.9-fold higher in HBE cells from patients homozygous for F508del and 2.5-fold higher in CFBE41o- cells than in non-CF cells.^151^ Therefore, they tested various well-known PARP-1 inhibitors for their effect on CFTR function and expression. Among them, the benzimidazole inhibitor ABT888 (Veliparib **27**, Figure 15)^152^ partially restored F508del-CFTR activity and trafficking in CFBE41o- cells at low concentrations (maximal inhibition observed at 1 nM). Similarly, treatment ex vivo of ileum tissues from CF mice with **27** partially rescued F508del-CFTR activity to 7% of WT levels and in vivo to 7.8% by measuring salivary secretion. Moreover, when PARP-1 activity was inhibited pharmacologically or by siRNA-mediated silencing, F508del-CFTR maturation was altered, with an increase in the fraction of the mature CFTR glycoform. In addition, the effect of PARP-1 inhibition was seen to be F508del-CFTR specific, as no improvement in the maturation of WT-CFTR was observed. Therefore, the same authors speculated that attenuation of PARP-1 activity could lower oxidative stress that is particularly high in CF and increase expression and folding of F508del-CFTR, at least partially, by altering PARylation of key members of CFTR folding interactome.^151^ However, further studies are required to uncover **27** mechanism of action in the context of CFTR proteostasis regulation.

In the same year, to diversify the collection of CFTR correctors previously discovered, the same research group screened a marine extract collection derived from South Pacific sponges using a bioassay-guided fractionation.^154^ With this method, they identified the class of latonduine heterocyclic compounds **28**–**30** (Figure 15),^153^ which corrected F508del-CFTR trafficking up to 45% of WT-CFTR surface expression. Among these alkaloids, latonduine A (**28**) strongly rescued F508del-CFTR misfolding in both BHK and CFBE41o- cells, and this result was confirmed in both ex vivo and in vivo studies in F508del-CFTR mice. Using pull-down experiments and MS studies, the authors identified PARP-1–5 as target proteins of **28**. Finally, combined treatment of **28** with other F508del-CFTR correctors, such as **2**, gave increased levels of correction, thus suggesting that **28** might define a novel class of potential CF therapeutics acting through PARP inhibition.^154^

In order to identify analogues of **28** with improved CFTR corrector potency and to understand the mechanism by which PARP inhibition improves F508del-CFTR trafficking, Thomas and co-workers synthesized a small set of analogues of **28**.^155^ The removal of the aminopyrimidine moiety and the replacement of the pyrrole with a phenyl ring led to the tetrahydrobenzoazepinone analogue MCG315 (**31**, Figure 15), a 10-fold more potent corrector than the parent compound, as evinced by short-circuit current measurements on CFBE41o- monolayers in Ussing Chamber assays. Moreover, enzyme inhibition assays showed that **28** and **31** in vitro behave as strong inhibitors of both PARP isozymes 3 and 16, perhaps through binding to the nicotinamide binding pocket, as evinced from molecular modeling studies. Further analysis showed that siRNA inhibition of both PARP-3 and PARP-16 resulted in a decrease in the concentration of **31** necessary for maximal F508del-CFTR rescue.^155^ Intriguingly, PARP-16 is an ER membrane-associated protein, which can ADP ribosylate the stress sensor IRE-1, ultimately triggering the activation of the unfolded protein response (UPR), whose role is to eliminate aberrant proteins.^156^ Therefore, in line with other works that showed that modulation of IRE-1 and UPR pathway can rescue F508del-CFTR trafficking,^157^**28** and its analogue **31** might trigger F508del-CFTR correction by inhibiting PARP-16-mediated UPR activation and by simultaneously inhibiting PARP-3.^155^

**Figure 16:**
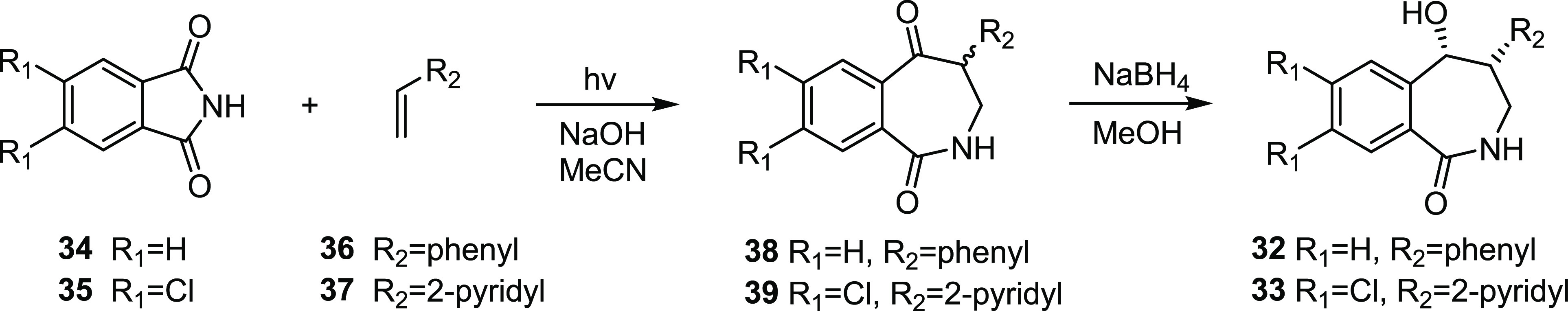
Synthesis of the two PARP-3 and PARP-16 selective inhibitors **32** and **33**.^158^

In order to confirm this proposed dual-target F508del-CFTR corrector mechanism of action of **28** and **31**, the same authors recently performed a chemical campaign around the tetrahydrobenzoazepinone scaffold to obtain compounds selective against PARP-3 or PARP-16 enzymes.^158^ Therefore, they reported the discovery of the two selective inhibitors **32** and **33** (Figure 16). Photochemical reaction of phthalimides **34** and **35** with alkenes **36** and **37** yielded the benzoazepinediones **38** and **39**, respectively, which were, in turn, reduced by NaBH_4_ to afford the corresponding alcohols **32** and **33** as racemic mixtures. Purification of the desired isomers followed by single-crystal X-ray diffraction and NMR analysis confirmed the *cis* configuration of (+/−) **32** and (+/−) **33**.

**Figure 17:**
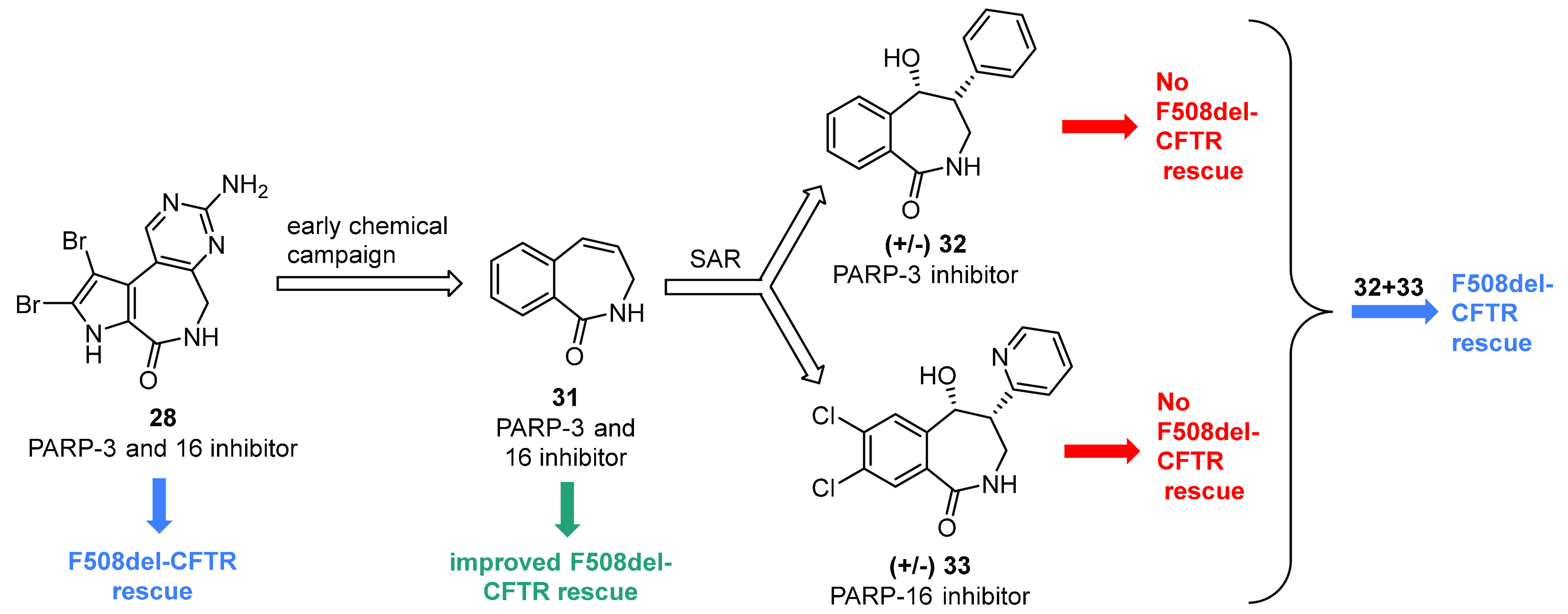
Latonduine A (**28**) optimization and strategy to prove that the PARP-mediated F508del-CFTR trafficking correction could be achieved by administrating either a single dual-target inhibitor (**28** or **31**) or a combination of two selective single-target inhibitors (**32** and **33**).^158^

In vitro evaluation of compounds ability to inhibit PARPs showed that **32** was a modestly potent PARP-3 selective inhibitor (IC_50_ = 3.1 μM) relative to PARP-16 (IC_50_ = 296.3 μM), whereas compound **33** displayed strong inhibition of PARP-16 (IC_50_ = 0.362 μM) with no significant effect on PARP-3 (IC_50_ = 74.1 μM). Interestingly, HTS and FMP cell-based assays showed that neither **32** nor **33** alone at either 1 or 10 μM elicited F508del-CFTR corrector activity, while the combination of these two selective inhibitors at both 1 or 10 μM each produced the same functional correction generated with an equal amount of **28** (Figure 17). These data strongly confirmed the authors’ hypothesis that the F508del-CFTR rescue exhibited by **28** and **31** could be caused by the dual-target simultaneous inhibition of PARP-3 and PARP-16.^158^ However, the mechanism of CFTR rescue enhanced by PARPi needs to be explained in more detail in order to anticipate possible side effects.

## 6 Targeting CFTR-Associated PDZ Domain Protein

The long-lasting PM expression of WT-CFTR depends on the endocytic trafficking events that occur at the cell surface, such as CFTR internalization by clathrin-mediated endocytosis (CME) and efficient recycling back from the endosomes to the PM.^159,160^ On the contrary, rescued F508del-CFTR shows a short PM half-life, due to increased endocytosis,^161^ selective ubiquitination by peripheral protein QC machineries, and rapid lysosome degradation of the mutant protein.^162^ Several protein partners regulate CFTR stability on the PM, among which PDZ domain-containing proteins (PDZ proteins) are most relevant.^163,164^ The C-terminus of CFTR binds two types of PDZ proteins: Na^+^/H^+^ exchanger regulatory factors 1 and 2 (NHERF-1 and -2) that work as scaffold proteins and stabilize CFTR on the PM by coupling it to the actin cytoskeleton^165−167^ and CFTR-associated ligand (CAL) that negatively regulates CFTR abundance by promoting its lysosomal degradation.^168^ Intriguingly, RNA interference-targeting CAL specifically increased cell-surface expression of F508del-CFTR by 4.4-fold and reduced CAL-mediated degradation,^169^ suggesting that selective inhibitors of the CAL-CFTR interaction could provide a novel generation of CFTR proteostasis regulators. The structure of CAL PDZ domain (CALP) bound to CFTR was solved by resolution NMR and showed interactions between the four C-terminal residues of CFTR peptide (residues Asp-Thr-Arg-Leu) and CALP.^170^ Madden and co-workers initially validated the possibility of selective PPIs disruption with the discovery of a peptidyl inhibitor of the CFTR-CAL interaction able to bind to CALP with high affinity and therefore hypothetically displace the natural binding partner CFTR.^171^ Their approach involved the synthesis of up to 6000 different cellulose-bond peptides through SPOT technology, with free C-terminal domains. Peptide screening and iterative optimization using substitutional analysis finally resulted in the identification of a decameric peptide inhibitor iCAL-36 (**40**, Table 1) with exhibited affinity of 22.6 ± 8.0 μM for CALP^172^ and no interaction with the NHERF PDZ domains, as determined by fluorescence polarization (FP) measurements and pull-down experiments.^171,173^ Remarkably, a control decameric C-terminal CFTR sequence (CFTR_10_, Table 1) exhibited interactions with the CAL PDZ domain (*K*_i_ = 390 μM) weaker than those exhibited by **40**.

**Table 1:** Sequences and Affinity Constants of Peptides Binding to the PDZ Domain of CAL

peptide	sequence^a^	*K*_i_ (μM)	ref
CFTR_10_	Thr-Glu-Glu-Glu-Val-Gln-Asp-Thr-Arg-Leu-OH	390 ± 20	(171)
iCAL36 (**40**)	Ala-Asn-Ser-Arg-Trp-Pro-Thr-Ser-Ile-Ile-OH	22.6 ± 8.0	(172)
kCAL01 (**41**)	Ac-Trp-Gln-Val-Thr-Arg-Val-OH	2.3 ± 0.2	(174)

a Ac = acetyl.

**Figure 18:**
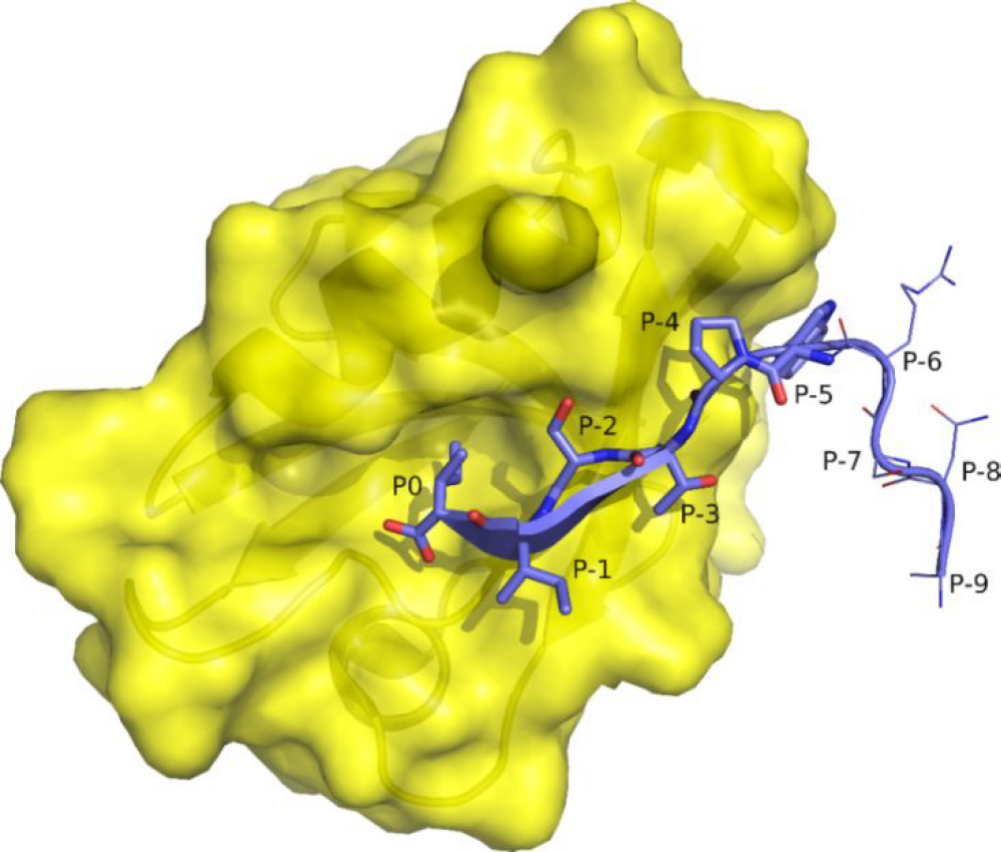
Interaction between PDZ peptide-binding domain (surface, yellow) and the decameric peptide iCAL36 (**40**) (stick, cyan) (PDB: 4E34).^172^

To visualize this new inhibitor functional effect on CFTR activity, a N-terminally fluorosceinated analogue of **40** (F*-**40**) was synthesized for Ussing Chamber assays. The treatment of CFBE bronchial epithelial cells from CF patients expressing F508del-CFTR with F*-**40** increased the half-life and the amount of apical F508del-CFTR channels, and this CFTR rescue effect was magnified when **40** was combined with the first-generation corrector **5**.^173^ New structural insights were obtained later with the determination of a high-resolution structure of CALP in complex with **40** (PDB ID: 4E34)^172^ and by chemically modifying side chains at different positions along the CALP binding cleft.^175^ This studies revealed that **40** could bind through canonical class 1 PDZ binding interactions, allowing the ligand C-terminal residue (P0) to form a critical interaction with the carboxylate-binding loop,^172^ while side chain interactions of residues P-1, P-3, P-4, and P-5 might be responsible for CALP affinity and specificity (Figure 18).^175^

To further expand their work on CAL/CFTR PPI disruption, Madden and co-workers used a new computational protein design algorithm (K*) to rationally develop a better binding-efficient competitive peptide CAL inhibitor.^174^ Using K* to calculate accurate predictions of peptide–CALP binding affinities, they screened up to 8000 hexameric C-terminal peptides from the HumLib library. The top-ranked 11 peptides predicted with the K* CAL-CFTR design were purchased from NEO BioScience, and their *K*_i_ values were determined using FP. All examined sequences showed high CAL affinity in the μM range, with kCAL01 (**41**) representing the tightest hexameric binder (Table 1; *K*_i_ = 2.3 ± 0.2 μM). Despite its smaller size, **41** yielded a higher affinity than the decamer **40** and a 170-fold stronger binding than natural CFTR C-terminus. Furthermore, **41** restored F508del-CFTR-mediated chloride efflux in CFBE cells in Ussing Chamber experiments, similarly to the previously available inhibitor F*-**40** or to the corrector **5**.^174^ Structure and energy landscape analysis of the crystal structure of **41**:CALP (PDB ID: 6OV7) showed that the tighter binding efficiency of **41** could stem from entropic effect at P0 and favorable substitutions at P-1 and P-4 with long polar and charged residues (from Ile and Pro of **40** to Arg and Gln of **41**, respectively).^176^

**Figure 19:**
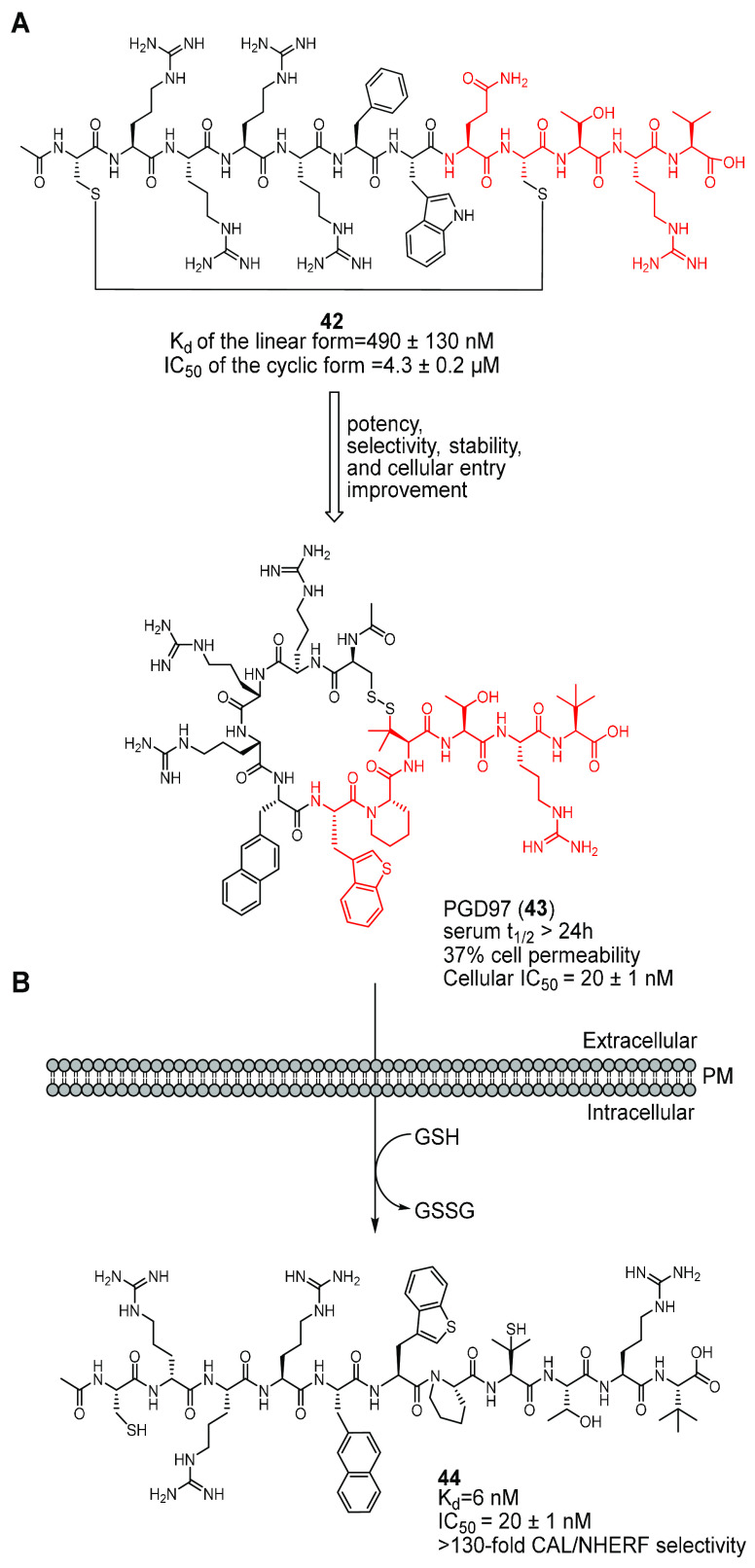
Development of disulfide-cyclized peptidyl inhibitors of the CAL-CFTR interaction from Dougherty et al.^177,178^ (A) Depiction of the SAR optimization of the peptide **42** to obtain PGD97 (**43**). The CPP sequence (in black) allows efficient cell permeation of the disulfide-cyclized conjugates, and the CAL binding sequence (in red) allows binding to CALP. (B) Strategy for cyclized peptide **43** cellular entry and conversion to the linear form **44**. When outside the cell, the CPP conjugate **43** is proteolytically stable and cell-permeable. Once inside the cytosol, **43** is reduced into its linear, biologically active form **44** by intracellular thiols, such as glutathione (GSH).

It is well-known that peptides present inherent limitations in metabolic stability and cell permeability that prevent their use as pharmacological treatment. To overcome these limitations, Pei and co-workers designed a disulfide-cyclized analogue of **41** by incorporating a short amphipathic Cys-Arg-Arg-Arg-Arg-Phe sequence (cell-permeating peptide, CPP) to its N-terminus and replacing Val at position P-3 with Cys to allow intramolecular disulfide bond formation (peptide **42**, Figure 19A).^177^ The obtained peptide was labeled with fluorescein isothiocyanate (FITC), and flow cytometry was used to demonstrate that **42** was readily cell-permeable and had a superior serum stability. Furthermore, fluorescence anisotropy (FA) analysis showed that only the reduced, linear form of peptide **42** could bind the CAL-PDZ domain with *K*_d_ of 490 ± 130 nM, whereas the cyclic form could not. Treatment of CFBE41o- cells with a combination of **42** and the corrector **2** increased the activity of F508del-CFTR by 77%. Therefore, the authors hypothesized that peptide **42** can exist as a disulfide cyclized form with improved proteolytic stability when outside the cell and, due to the CPP motif, can show high cell permeability, too. Upon entering the cell, intracellular thiols convert **42** into its linear form, which can expose the CAL binding sequence for efficient displacement of the CFTR-CAL interaction. This effect may be responsible for the increase of F508del-CFTR stability, through hypothetical reduction of lysosome-mediated degradation of the mutant protein.^177^

**Figure 20:**
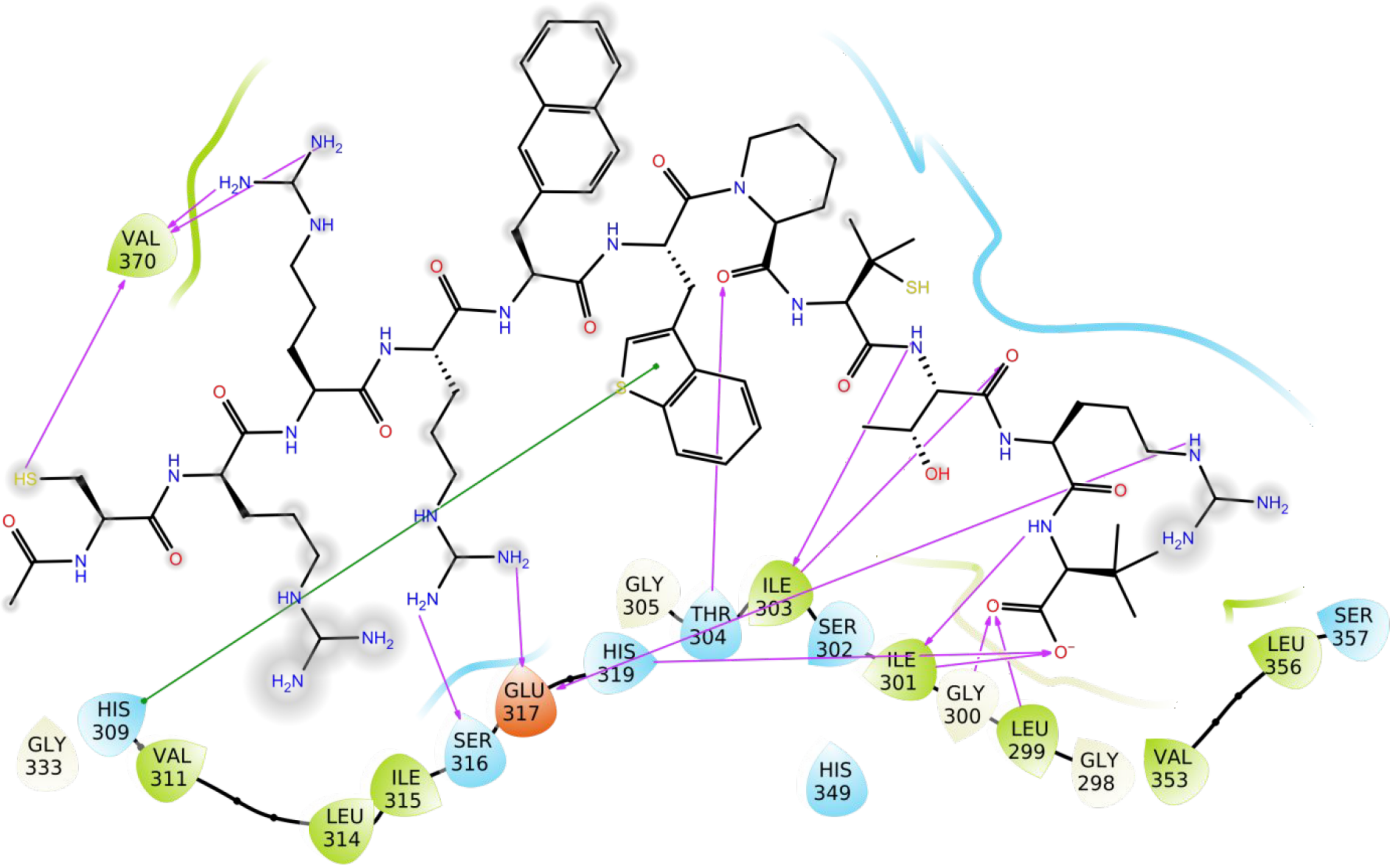
Diagram showing the key interactions between peptide **44** and the CFTR associated ligand (CAL) PDZ domain. Pink arrows indicate hydrogen bonds, and a green line indicates π–π stacking interaction. PDB from Dougherty et al.^178^

To obtain great improvement of peptide **42** potency, selectivity, and pharmacokinetic properties, the same authors recently performed a modeling-guided medicinal chemistry campaign through in silico binding evaluation of a library of peptide analogues, followed by the synthesis and FP-based competition assay of the sequences containing the best residues.^178^ First, they focused on enhancing CAL binding efficacy. With this strategy, they selected *tert*-butyl-l-alanine (Tle) as the P0 residue, which increased the binding affinity by 2.7-fold and proteolytic stability due to its bulky *tert*-butyl side chain. At the P-3 position, l-penicillamine (Pen) was incorporated, yielding a more conformationally defined disulfide bond, whereas at P-6 Phe was replaced with a larger hydrophobic 3-(2-naphthyl)-l-alanine (2-Nal), resulting in a 5-fold increase in CAL affinity. Concerning the CPP sequence, in order to optimize cell permeability and proteolytic stability, the number of arginine residues was reduced from four to three, and at the P-9 position a d-arginine was incorporated, yielding higher cytosolic entry efficacy as determined by flow cytometry. To further enhance peptide **42** CAL selectivity over NHERF, Gln at P-4 and Trp at P-5 were replaced by pipecolic acid (Pip) and 3-(3-benzothienyl)-l-alanine (Bta), respectively. All of these modifications ultimately produced the disulfide-cyclized peptide PGD97 (**43**, Figure 19A), which showed great cellular entry efficacy and high stability in human serum compared to the parent peptide **42**. Furthermore, the linear form of **43**, peptide **44** (Figure 19B), was highly potent and selective, with *K*_d_ = 6 nM and ≥130-fold selectivity for CALP vs NHERF. To gain insight about the structural basis of the exceptional binding affinity of peptide **44**, the authors analyzed its predicted binding mode with CALP (Figure 20). In particular, the C-terminal carboxylate of Tle (P0) could form key hydrogen bonds with the backbone amides of Leu299, Gly300, and Ile301, while the *tert*-butyl side chain of the same residue could interact with an adjacent hydrophobic area. Instead, Pip at P-4 might facilitate the peptide to assume an optimal conformation that might position the benzothienyl ring of Bta at P-5 for a critical π–π interaction with His309. Biological evaluation of **43** indicated that it strongly increased the surface expression, stability, and function of F508del-CFTR in CFBE41o- cells. Furthermore, in CF-patient-derived HBE cells, **43** increased F508del-CFTR ion channel activity by ∼3-fold (EC_50_ ∼ 10 nM) and further potentiated the therapeutic effect of the known corrector **3** by ∼2-fold.^178^ All of these data demonstrated that the authors successfully developed a drug-like cyclized peptidyl molecule as a potent, selective, and with high proteolytic stability inhibitor of CAL-CFTR PPI. This creates interest in further optimizing **43** for clinical trial evaluations and in developing other peptidyl inhibitors to rescue F508del-CFTR PM stability.

**Figure 21:**
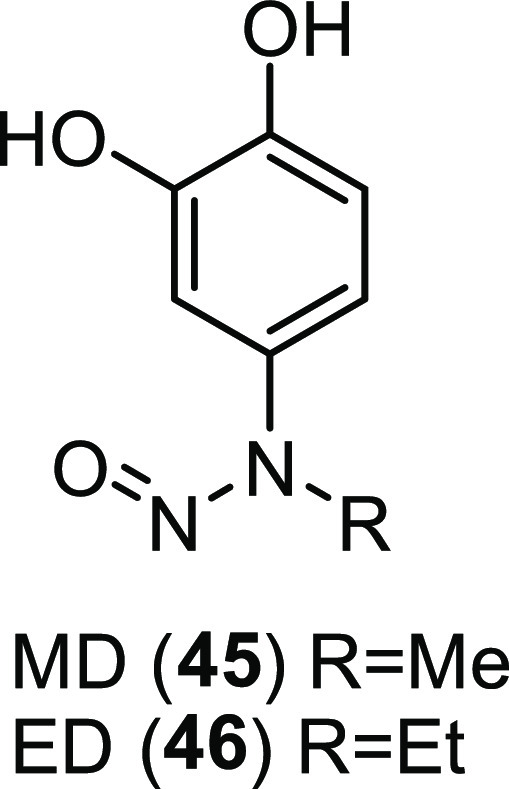
Chemical structures of the catecholic compounds MD (**45**) and ED (**46**).

Noteworthy, there have also been efforts to develop small molecule inhibitors of this PPI. Madden and co-workers performed a comparative HTS using peptide **40** as control and either FRET or AS proximity assays as primary screen.^179^ Of the 3161 tested chemical compounds of the St. Jude bioactive collection, 12 hits were identified with both approaches, and among them, HSQC footprints of the CALP identified two compounds giving residue-specific chemical-shift perturbations. One of them, the methyl-3,4-dephostatin MD (**45**, Figure 21) did not exhibit cytotoxic and cytostatic effects when applied to F508del-CFBE monolayers, but unfortunately, it failed to increase F508del-CFTR chloride current in Ussing Chamber experiments when tested in the same cell model. Crystallographic and NMR studies showed that **45** could interact in a distinct site than the canonical peptide-binding domain of CALP. Further investigations revealed that the catechol **45** and its close analogue ethyl-3,4-dephostatin ED (**46**, Figure 21) might function by covalently binding to CAL by forming a cysteine adduct. Therefore, despite **45** and **46** representing the first example of small molecules able to regulate PDZ-CFTR interaction, their utility as drug scaffolds remains limited because of their ability to covalently modify proteins. Moreover, **45** and **46** are likely to be pan-interference compounds (PAINS)^180^ and exhibited involvement in several regulation pathways, which might lead to undesired off-target effects.^179,181−183^

## 7 Additional Pharmacological Strategies under Investigation

### 7.1 Restoring Defective Autophagy

**Figure 22:**
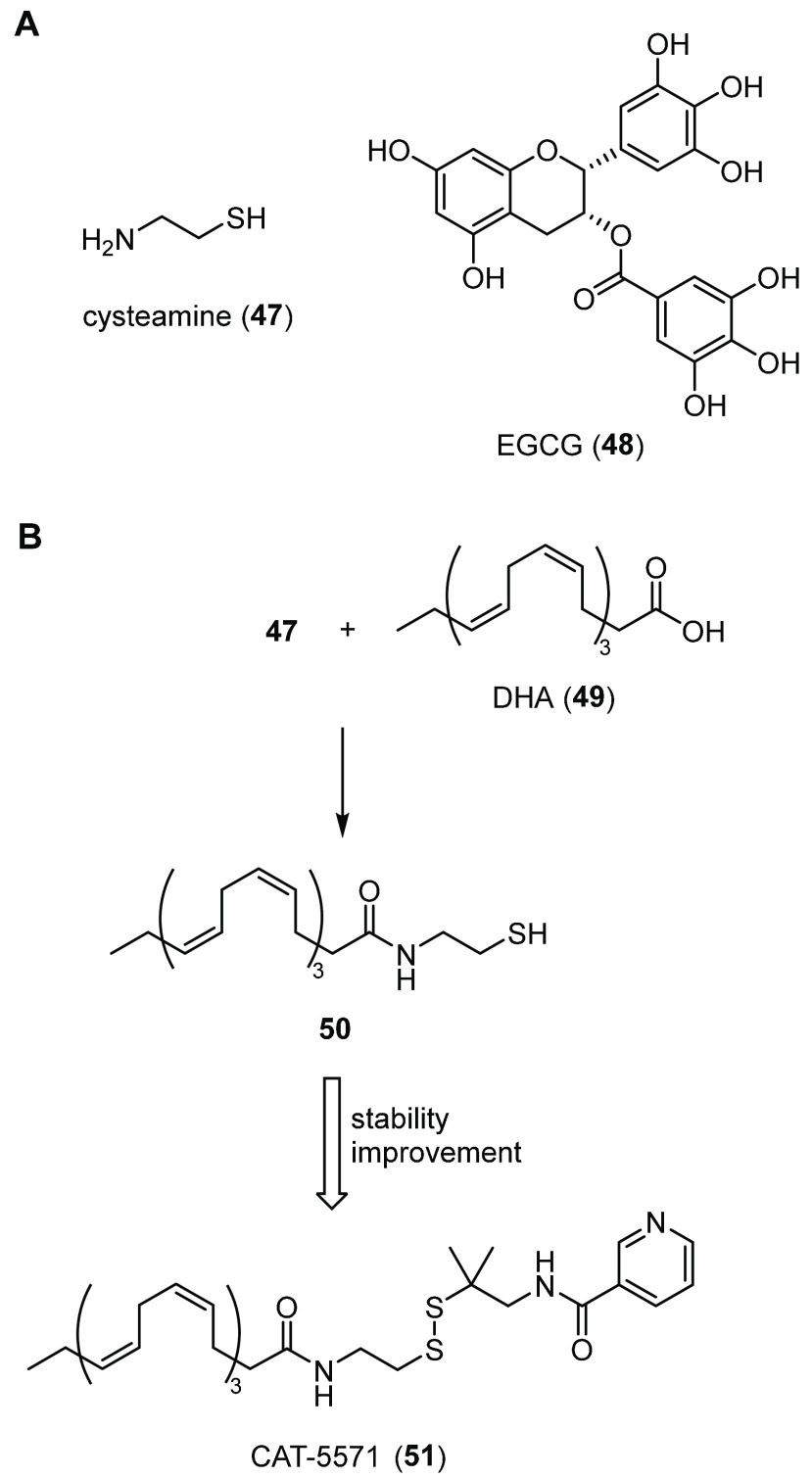
Autophagy activators in study as F508del-CFTR proteostasis regulators. (A) Chemical structures of cysteamine (**47**) and EGCG (**48**). (B) Strategy for **47** potency and stability optimization to obtain CAT-5571 (**51**).^193^

There is an emerging interest for autophagy modulating compounds in controlling pathogenesis of CF disease,^184^ although this field of research remains controversial for many reasons. Raia and co-workers have been some of the major exponents of the research aimed at finding autophagy inducers as CF therapeutics. They initially demonstrated that human CF airways are autophagy-deficient, a condition that leads to decreased clearance of aggresomes (misfolded proteins aggregates).^185,186^ Autophagy is a key process in cellular clearance of protein aggregates and removal of ROS sources.^187^ Dysfunctional F508del-CFTR is believed to induce the generation of ROS that lead to an increase of the activity of profibrotic tissue transglutaminase 2 (TG2).^186^ The increase in TG2 activity, in turn, drives the sequestration of beclin-1 and the corresponding accumulation of p62, two key proteins in autophagosome formation.^188^ These events trap misfolded CFTR at the ER level, thus leading to rapid F508del-CFTR degradation and decreased trafficking to the PM. To investigate whether the restoration of autophagy could revert these sequential events and allow rescue of F508del-CFTR trafficking, the same authors showed that, by overexpressing beclin-1 or knocking down p62, the level of F508del-CFTR at the cell surface increased. In addition, the TG2 inhibitor cysteamine (**47**, Figure 22A), already approved for the treatment of orphan disease cystinosis, had similar results, thus partially restoring in vivo expression of beclin-1 and slightly increasing PM expression of F508del-CFTR at a concentration of 250 μM in nasal epithelial cells from CF patients.^186,189^ Although the effect of **47** was modest and a high concentration was required to activate autophagy, the authors moved forward by performing a phase 2 pilot clinical study with 10 F508del-CFTR homozygous patients. Their results showed that the combination of **47** and the natural epigallocatechin 3-gallate (EGCG, **48**, Figure 22A)^190^ activated autophagy and improved mutant CFTR function. They speculated that the addition of **48** could modulate a different related pathway, such as the protein kinase CK2 involved in proteolytic degradation of mutant CFTR. Therefore, by inhibiting CK2, **48** might increase F508del-CFTR stability at the PM after **47**-mediated rescue.^189,191,192^ However, to our knowledge, the effect of **47** alone or in combination with **48** on F508del-CFTR rescue has not been confirmed by other researchers to date. Indeed, three different research groups could not detect F508del-CFTR functional rescue using the same concentration of **47** in well-differentiated HBE cells, and among them, our group even reported deleterious effects on CFTR expression and activity after treatment with **47**/**48** combination.^193−195^ Similarly, despite promising initial data in cell and animal models, **48** failed in clinical trials for different proteinopathies.^196^ One of the reasons for its negative outcomes could be the lack of a clear understanding of the mechanism of action for **48** and its critical molecular targets.^196^ These results raise some concern about the activity of the autophagy modulator **47** and its combination with the antioxidant **48** and call for other confirmational evidence. Furthermore, **47** administration comes with many problems, such as the low potency, unpleasant thiol smell and taste, and short half-life. The chronic treatment with high doses of **47** is therefore not feasible and can lead to undesirable off-target and side effects.

In order to find a more potent, yet safe and effective autophagy activator for further proof-of-concept studies, Liu and co-workers used their expertise in fatty acid conjugates to synthesize a covalent conjugate of **47** and docosahexaenoic acid (DHA, **49**, Figure 22B),^193^ a ω-3 fatty acid that previously demonstrated to induce autophagy.^197,198^ With this strategy, the authors believed that the conjugate **50** (Figure 22B) could allow delivery of equimolar concentrations of **47** and **49** inside the cells, thus enabling synergism of the two bioactive components in terms of autophagy activation. Further biological experiments showed that **50** could increase beclin-1 levels and activate autophagy at concentrations (3 μM) lower than those of **47** in primary homozygous F508del-CFTR HBE cells. Interestingly, the individual components (**47** and **49**) either alone or in combination were not able to replicate the same level of activation, even at concentration of 250 μM. When compound **50** was used in triple combination with the potentiator **1** and the corrector **2**, an additive effect was obtained, as evinced from immunoblot and F508del-CFTR chloride conductance assays. However, the authors reported solubility issues of **50** at concentrations greater than 3 μM, and a wide range of responses were observed depending on the primary CF-HBE cells used. Furthermore, conjugate **50** showed intrinsic instability, perhaps due to the sulfhydryl moiety. To enhance the stability of **50**, the authors synthesized a small set of analogues, by converting the sulfhydryl group to an amide or a disulfide bound carrying different functionalities. Among them, conjugate CAT-5571 (**51**, Figure 22B) with a sterically hindered methylpropylnicotinamide moiety adjacent to the disulfide bound showed improved stability in rat, mouse, dog, and human plasma and was orally available due to a self-emulsifying dispersion formulation.^193^ Moreover, the authors reasoned that the new potent and bioavailable autophagy activator **51** could have a significant impact on intracellular clearance of bacteria, too, which is highly recommendable in CF patients that are subjected to chronic lung bacterial infections. Therefore, **51** has the characteristics for representing a potential new proteostasis regulator with multiple therapeutic effects and for that was included in preclinical studies at Catabasis Pharmaceuticals. Treatment with **51** in vitro and in vivo efficiently restored autophagy and caused a significant reduction in the intracellular bacterial load of *Pseudomona aeruginosa* and *Burkholderia cenocepacia*.^199^ In addition, **51** was able to enhance cell-surface trafficking and function of F508del-CFTR in combination with **1** and **2**.^199^ At present, Catabasis is conducting a preclinical study in collaboration with the Bill & Melinda Gates Medical Research Institute to evaluate **51** as a potential oral therapy to promote autophagy and clear persistent lung infections in patients with both drug-sensitive and drug-resistant tuberculosis (TB).^200^

Recently, Romani et al. investigated the effect on F508del-CFTR rescue of thymosin alpha 1 (Tα1, **52**), a well-known polypeptide in immunotherapy with sequence Ser-Asp-Ala-Ala-Val-Asp-Thr-Ser-Ser-Glu-Ile-Thr-Thr-Lys-Asp-Leu-Lys-Glu-Lys-Lys-Glu-Val-Val-Glu-Glu-Ala-Glu-Asn.^201^**52** is commercialized under the trade name of Zadaxin for the treatment of viral infections, immunodeficiencies, malignancies, and HIV/AIDS.^202^ Its mechanism of action involves the induction of the immunoregulatory enzyme indoleamine 2,3-dioxygenase 1 (IDO1) in the bronchial epithelium, thus potentiating immune tolerance in the lung, reducing inflammation and activating autophagy.^203^ In accordance with their previous works on **47**, the authors believed that, through promoting autophagy, **52** could affect trafficking and expression of F508del-CFTR. Therefore, they performed in vitro and in vivo experiments reporting that **52** could suppress inflammation and at the same time rescue F508del-CFTR maturation, stability, and activity in CFBE41o- cells, primary HBE cells, and in a CF murine model.^201^ Furthermore, they reported that **52** also increased the chloride current of the calcium-activated chloride channel (CaCC) in three of five patients examined, thus promoting a compensatory chloride secretion. For all of these reasons, the authors proposed **52** as a single-molecule-based therapy for CF patients with F508del.^201,204^ Since then, our group along with five other independent CF research groups have been trying to reproduce these results but failed to obtain any correction of F508del-CFTR or activation of CaCC in several bronchial cell cultures and using different measurement protocols.^195,205,206^ All of these results do not exclude the beneficial effects of **52** on the immune system and inflammation but call into question its use as a CFTR proteostasis regulator.

**Figure 23:**
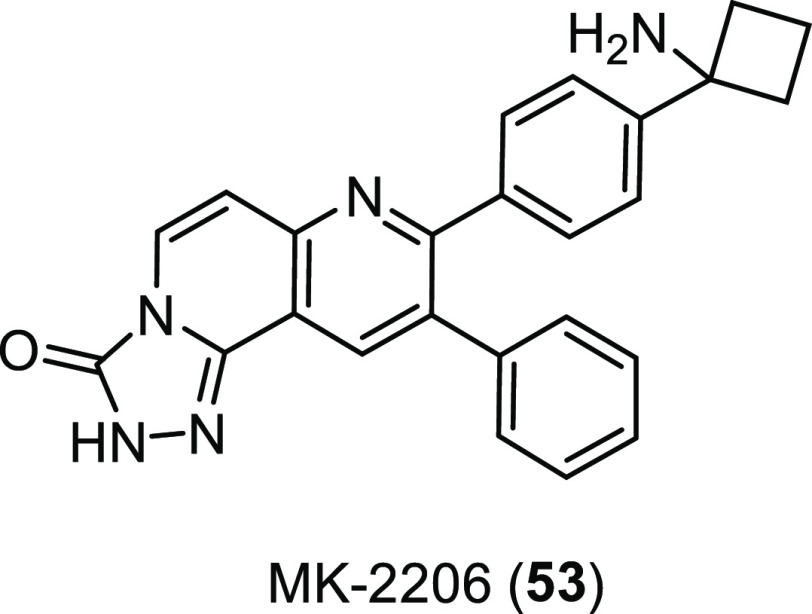
Chemical structure of the mTOR/Akt inhibitor MK-2206 (**53**).

A further noteworthy study in this field is represented by the work of Coppinger and co-workers. Using protein interaction profiling and global bioinformatics analysis, these authors identified PI3K/Akt/mTOR as an additional signaling pathway involved in autophagy that could be targeted to obtain F508del-CFTR rescue.^207^ This axis has a central role in cell growth and stress response,^208,209^ with the serine/threonine kinase rapamycin (mTOR) tightly regulating autophagosome formation and Akt directly phosphorylating beclin-1.^210,211^ The authors’ analysis showed that mTOR activity was up-regulated in F508del CFBE41o- cells and therefore hypothesized that small molecule inhibitors of the PI3K/Akt/mTOR complex could be useful to promote F508del-CFTR stability and function. For that, a small set of six known inhibitors targeting different components of the pathway were purchased from commercial sources and tested for their effect on F508del-CFTR and on defective autophagy restoration in F508del CFBE41o- cells. Among them, the mTOR/Akt inhibitor MK-2206 (**53**, Figure 23) displayed the highest increase in mutant CFTR maturation, stability, and expression and strong induction of autophagy. Furthermore, PI3K/Akt/mTOR inhibition also decreased the levels of BAG3, a cochaperone of the Hsp70/Hsc70 complex involved in the autophagic degradation of misfolded protein aggregates. Thus, the authors speculated that the mechanism of action of **53** might involve the activation of autophagy through inhibition of both PI3K/Akt/mTOR and BAG3 axes, which, in turn, could decrease the sequestration of mutant CFTR into aggregates and lead to channel rescue to the PM. However, PI3K/Akt/mTOR inhibitors are associated with several off-targets and side effects, such as severe hepatotoxicity and pneumonitis, which have restricted their application and clinical significance.^212,213^ This limits possible use of **53** in CF therapy and suggests that further studies are needed to address its utility as a proteostasis regulator scaffold.

### 7.2 Targeting Kinases

**Figure 24:**
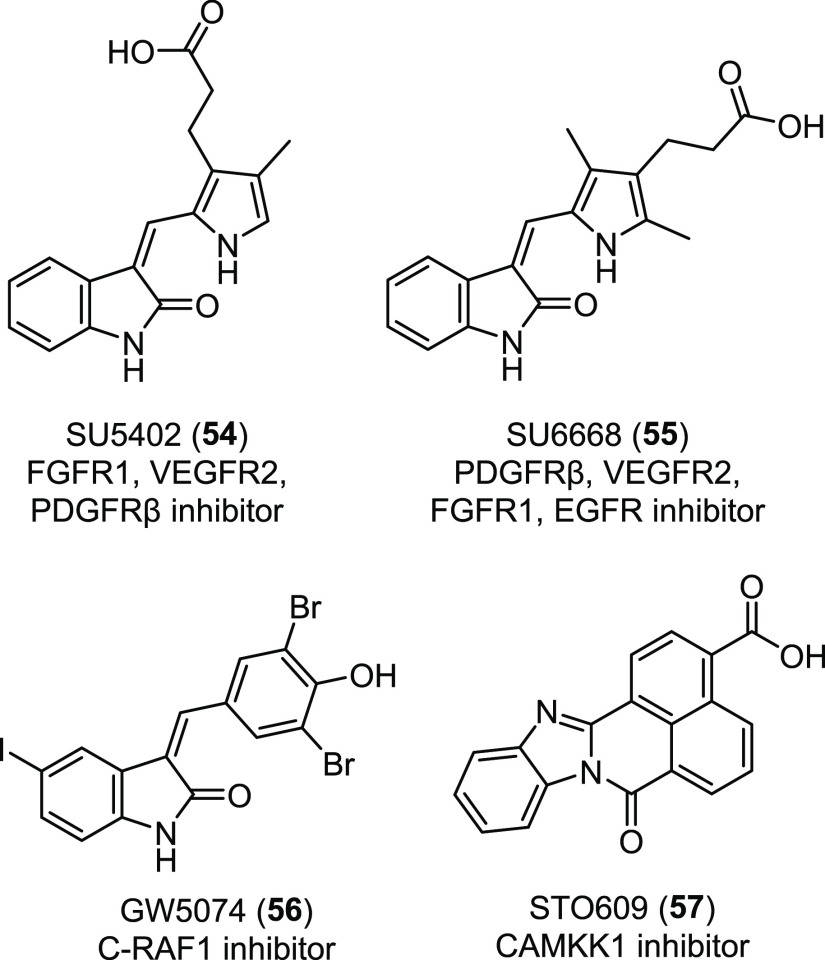
Chemical structures of some of the hit kinase inhibitors identified by Rotin et al. and Bruchez et al. that showed an enhancement of F508del-CFTR rescue.^214,216^

Protein kinases are involved in several cellular pathways and processes, including CFTR degradation, where they presumably affect specific chaperones that normally control the maturation of the protein (e.g., Hsp70/Hsc70, Hsp90 chaperone complexes). Rotin and co-workers have been working for many years on the identification of the kinases that could play a role in CFTR processing and if their inhibition could lead to rescue of F508del-CFTR function. First, using a high-content screening protocol, the authors performed a kinase inhibitor screen with a library of 231 compounds, including FDA-approved drugs or compounds in clinical trials for the treatment of different diseases, mainly cancer and inflammation.^214^ The kinase inhibitors were purchased from different vendors or synthesized when not commercially available and were chosen to target as many kinases as possible. In vitro treatment with several compounds resulted in an increase of the F508del-CFTR activity. The 41 inhibitors showing the higher F508del-CFTR rescue were further validated by biochemical and electrophysiological techniques in different cell types, including primary HBE cells from CF patients. The results showed that several compounds increased F508del-CFTR maturation, expression, and activity at nanomolar concentrations. In particular, inhibitors of the receptor Tyr kinases (FGFRs, VEGFR, and PDGFR), such as the indolinone SU5402 (**54**, Figure 24) and its analogue SU6668 (**55**, Figure 24), already in clinical trials mainly for cancer therapy, showed a robust rescue of the mutant CFTR channel.^214^ However, how the identified kinase inhibitors could possibly rescue F508del-CFTR needed further investigations.

In order to complement the small molecule screen, in 2015, the same authors performed an additional kinome screen with a library of endoribonuclease-prepared short interfering RNAs (esiRNA)-targeting 759 different kinases.^157^ With this strategy, several genes were identified eliciting significant F508del-CFTR rescue when knocked down, including those of the previously reported FGFR pathway. Indeed, silencing of FGFRs and their downstream proteins further validated the involvement of this signaling cascade in F508del-CFTR trafficking and maturation. Treatment of F508del-CFTR mice or intestinal organoids generated from the same animals with the FGFR1 inhibitor **54** led to a promising rescue of the mutant channel. In addition, in vitro combination of **54** with the corrector **2** resulted in an additive effect, suggesting that the two bioactive molecules might have different mechanisms of action. Indeed, the authors claimed that FGFR inhibition by **54** might regulate different chaperones involved in F508del-CFTR maturation, as evinced from chaperone array analysis.^157^ However, **54** exhibits significant off-target activities, such as the inhibition of several tyrosine kinases different from FGFR1.^215^ Thus, **54** is far from having a possible therapeutic value for the treatment of CF, and the development of more specific and potent analogues is pivotal for further investigations.

More recently, Bruchez and co-workers used an innovative HTS methodology to identify druggable kinases able to enhance F508del-CFTR trafficking and stability at the cell surface.^216^ Indeed, they previously developed and validated a fluorogen-activating protein (FAP)-based platform for quick and selective detection of F508del-CFTR surface expression and overall protein content onto the PM.^217,218^ Using this technology, the authors performed the screening of the siRNA kinase library (Dharmacon ON-TARGETplus SMARTpool) targeting 715 different kinases, with or without corrector **2** co-treatment.^216^ In brief, to determine the ratio of exposed F508del-CFTR channels over the total protein expressed, the authors used different types of fluorogens to label first the surface and then the intracellular CFTRs present in HEK293 cells expressing FAP-F508del-CFTR. In particular, the malachite green sulfonated analogue MG-B-Tau was used for CFTR surface labeling, while for intracellular CFTRs, a novel type of cell-permeable dye was synthesized, the *n*-butylamine MGnBu. When the FAP protein fused to F508del-CFTR binds to the fluorogens, the dye becomes fluorescent and can be revealed. Interestingly, with this technology, several target kinases were identified, whose suppression showed an increase in F508del-CFTR surface density in combination with treatment with **2**. Among them, kinases CAMKK1 and RAF1 were the most promising targets. Further validation with selective RAF1 and CAMKK1 inhibitors, GW5074 (**56**, Figure 24) and STO609 (**57**, Figure 24), respectively, showed significant F508del-CFTR rescue compared to **2** treatment alone, as measured by flow cytometry. These results confirmed the sensitivity and robustness of the high-throughput methodology proposed by the authors, paving the way for its application to different cellular targets in future. Although the identification of druggable targets maintains a role of primary importance, fewer efforts have been done to date to discover new drug-like molecules acting as kinase inhibitors able to rescue mutant CFTR defects. Once this gap will be filled with new proof-of-concept studies, we believe that a better understanding of the role and consequences of these kinases inhibition will be available and may guide new CF research projects.

### 7.3 Targeting Histone Deacetylases HDACs

Histone acetyl transferases (HATs) and deacetylases (HDACs) have been extensively studied for their role in modulating transcriptional events implicated in several human diseases.^219^ These enzymes catalyze the post-translational addition or removal of acetyl functional groups to histones, transcription factors, and other cytosolic components (e.g., the chaperon Hsp90), thus regulating their activity.^219,220^ Histone deacetylase inhibitors (HDACi) serve as transcriptional activators, and they have been proven to be beneficial in many pathological conditions, as reported in several recent reviews.^221−223^ Mammalian genome encodes 11 HDACs categorized into four different classes, but the precise role of each isoform in cellular function is not yet completely understood.^224^ HDACi have represented useful tools to help clarify these issues, as they inhibit different HDACs mostly by interacting with their catalytic sites, leading to decreased deacetylation of histones and proteins. Moreover, HDACi could be potentially useful to influence misfolding protein maturation and function, such as F508del-CFTR, through either activating transcription of CFTR-related genes or modulating the activity of proteins involved in CFTR processing.

**Figure 25:**
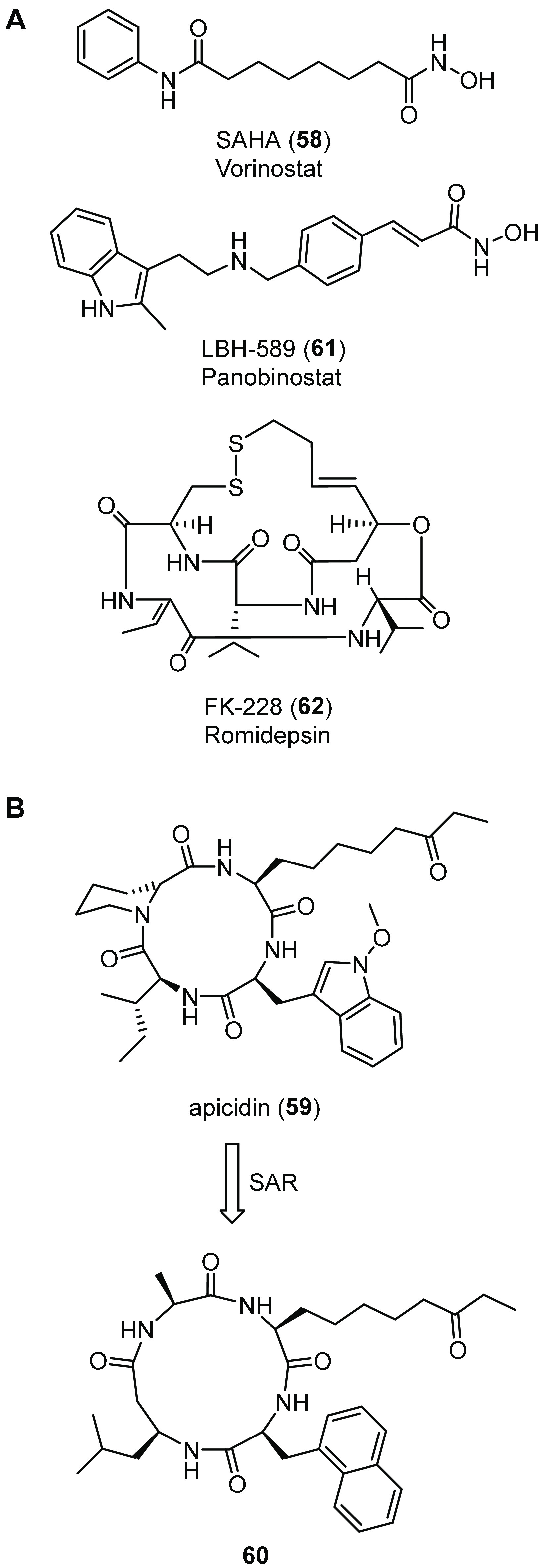
HDAC inhibitors in study as F508del-CFTR correctors. (A) Chemical structures of the known inhibitors SAHA (**58**), LBH-589 (**61**), and FK-228 (**62**). (B) Strategy for apicidin (**59**) F508del-CFTR corrector efficacy optimization to obtain the cyclic tetrapeptide **60**.^229^

Balch and co-workers showed that the suberoylanilide hydroxamic acid (SAHA, trade name Vorinostat, **58**, Figure 25A), the first FDA-approved HDACi for T-cell lymphoma treatment,^225^ yielded a notable correction of F508del-CFTR trafficking defect in the human lung CFBE41o- cell line.^226^ To identify the specific target of **58**, siRNA screening of several types of HDACs was performed, revealing that HDAC7 could be the key enzyme involved in **58**-mediated F508del-CFTR trafficking and activity rescue.^226^ Thus, the authors claimed that HDAC7 could be a viable pharmacological target for mutant CFTR correction in CF, even though **58** showed a very modest increase of F508del-CFTR activity in primary HBE cells. Hence, our group^227^ along with Bergougnoux et al.^228^ independently reported that **58** was not able to increase F508del-CFTR maturation and chloride secretion in primary airway epithelial cells from CF patients^227^ or in an ex vivo model of differentiated nasal cells obtained by scraping from CF patients.^228^ These different observed effects on the F508del-CFTR chloride channel could be explained by alterations in the baseline chromatin state between primary bronchial epithelial cells and immortalized cell lines. Therefore, these results suggest that the activity of **58** on F508del-CFTR rescue might have been overestimated from Balch and co-workers and call into question its efficacy and potential use for CF treatment.

Balch and co-workers further expanded their research on CFTR proteostasis regulators with an epigenetic mechanism by focusing on synthetic conformationally biased cyclic tetrapeptides related to the natural compound apicidin (**59**, Figure 25B), a known HDAC inhibitor.^229^ Cyclic tetrapeptide inhibitors are characterized by the presence of functionally critical Zn^2+^-coordinating chains containing terminal α,β-epoxyketone, ethylketone, amide, or carboxylic acid. These unnatural amino acids can be considered isosteres of acetylated Lys and therefore may interact with the HDAC zinc-binding domain by mimicking an acetylated Lys residue of a substrate protein.^230^ For this property, cyclic tetrapeptides and pseudotetrapeptides have been extensively studied for their strong HDAC inhibition profile.^230−232^ Indeed, the authors screened for their ability to rescue F508del-CFTR trafficking a library of cyclic tetrapeptide HDAC inhibitors designed to cover a broad range of pharmacophore configurations. The compounds of interest were synthesized by varying each one of the four amino acids, through amide alkylation, addition of side chain functionalities or zinc coordinating moieties, and introduction of β-amino acids or triazoles in the backbone. Among them, the 13-membered ring tetrapeptide **60** (Figure 25B) showed efficacy as the F508del-CFTR corrector higher than that of the parent apicidin **59** and compared to the previously evaluated inhibitor **58**, with levels of mutant CFTR rescue of nearly 40%. However, full HDAC inhibition profiling showed that **58** was a more potent and broad HDACs inhibitor than compound **60**, although **60** was a better F508del-CFTR corrector compared to **58**.^229^ From these results, the relationship between HDAC inhibition and F508del-CFTR rescue to the cell surface appears questionable, although cyclic tetrapeptide **60** may represent a novel type of CFTR corrector and further structure–activity optimizations may be explored in the future.

More recently, Balch and co-workers examined the impact of three additional FDA-approved HDACi on F508del-CFTR trafficking and function rescue in F508del-CFBE cells and in primary HBE cells from F508del homozygous patients.^233^ Two of the tested compounds, the indole LBH-589 (trade name Panobinostat, **61**, Figure 25A) and the cyclic peptide derivative FK-228 (trade name Romidepsin, **62**, Figure 25A) restored the functionality and PM expression of the mutant channel, and their effects were synergized with the corrector **2**. Interestingly, the authors further evaluated the effects of **61** and **62** on the rescue of additional types of CFTR mutants carrying less common class II and class III variants, which are responsible for differential onset and progression of CF disease. Although the two molecules gave different responses with respect to the CFTR variants taken into consideration, they could provide functional correction of the mutant proteins, both alone and in combination with **2**. Thus, the authors speculated that, by globally modulating the expression or activation of multiple cellular pathways, HDACi might facilitate the folding of different CFTR variants.^233^ Taken all together, HDAC inhibition remains a promising and questionable field of interest in CF research and ought to be further investigated by other independent researchers.

## 8 Challenges and Opportunities in Developing New Proteostasis Regulators

To date, numerous potential proteostasis regulator drug targets have been identified through genetic screening, proteomic and interactomic studies, and combinational approaches.^15,48−51^ However, this has yet to correlate with improved medicines in the clinic, with only the amplifier **8** reaching early clinical trials and the autophagy activator **51** being under preclinical evaluation.

From this overview, it appears that proteostasis regulator effects are additive with other correctors and therefore are expected to have a higher therapeutic ceiling and expand pharmacological treatment applicability to CF patients bearing mutations poorly responsive to already developed modulators. In particular, the most promising proteostasis regulators are those showing additive effects over the triple combination drug (Trikafta), which is by far the most effective treatment available—at the moment—for CF patients bearing eligible genotypes. However, most proteostasis regulators have been discovered in the pre-**4** era, thus their efficacy in combination with approved CFTR modulators has been evaluated mainly toward **2** only. Investigating the efficacy of proteostasis modulators in combination with **4**/**3** as well as the identification of those more effective on mutations poorly responsive to approved drugs could be helpful to prioritize them for further development.

Few research groups undertook the challenging path of evaluating novel proteostasis regulators on CF-causing mutations other than F508del. Perhaps this is due to differences in the CFTR interacting networks among the various CFTR genotypes, which may be responsible for different responses to proteostasis regulators treatment. Nevertheless, a possible use of such approaches to rescue less common CFTR mutants has much potential for CF, meeting a need that the CFTR pharmacotherapy from the previous 20 years has struggled to fill.

One of the largest barriers to the development of more drugs acting as CFTR proteostasis regulators is the complexity of CFTR regulome. For example, some target proteins may have pleiotropic effects on CFTR processing, such as the Hsc/Hsp70 and Hsp90 complexes that are implicated in both degradation and folding of mutant CFTR.^66^ Therefore, targeting these pathways may fall into the risk of unbalancing the delicate processes of CFTR biogenesis and rescue. Furthermore, both Hsc70 and Hsp90 systems have a crucial role in cell signaling and protein homeostasis, and therefore, their pharmacological manipulation hides many challenges at first glance, with many opportunities for toxicity. Many research groups and companies have studied Hsp90 and Hsc70 inhibitors with successful results over the past few decades.^99,234^ Taking as example Hsp90 inhibitors, most of the clinical trials that were initiated have been terminated due to lack of efficacy and severe off-target effects such as hepatotoxicity and ocular toxicity. Consequently, none has been approved by FDA until now.^235,236^ Additionally, Hsp90 and Hsc70 chaperone systems function together as a multiprotein dynamic complex.^237^ For that, the intervention with PPI inhibitors is complicated due to the highly dynamic interactions between Hsp90/Hsc70 and their cochaperones.

Other strategies under investigation, such as targeting E1 or DUBs, are a relatively nonspecific way to achieve mutant CFTR rescue and, for this reason, may not be amenable to therapeutic development. Indeed, inhibiting E1 might block the degradation of all proteins targeted by the UPS, leading to undesirable off-target effects. Additionally, the alteration of the ubiquitination/deubiquitination process might lead to the dysregulation of those biological networks in which ubiquitination plays an important regulatory role. Conversely, given the selectivity of ubiquitination provided by E3s, targeting RNF5 may overcome the specificity issues encountered with E1 inhibitors and DUB activators. Thus, inhibiting RNF5 ligase is expected to affect only a limited set of substrates, leading to fewer side effects. However, RNF5 regulates the degradation of proteins involved in different cellular process, such as autophagy,^238^ inflammation,^239^ cell migration,^240^ and innate antiviral response,^241^ with possible pathophysiological implications. For that, the concept of inhibiting this E3 ligase to treat CF remains controversial, and further investigations are required to consider it as potential therapeutic.

Concerning CFTR stabilization on the PM, difficulties may arise due to the generally low binding selectivity of CFTR-associated PDZ domain proteins, such as CAL.^242^ PDZ proteins play a crucial role in several molecular and physiological pathways, such as signal transduction, cell polarity, cell adhesion, and protein trafficking, which have made PDZ proteins very attractive targets for drug discovery through the years.^243^ However, under this light, selectivity in targeting CAL appears to be a difficult but crucial task for a successful CF therapy, in order to decrease cross-reactivity and improve efficacy.

Some candidate proteostasis regulators, such as PARPi or HDACi, still have an unknown or under investigation mechanism of F508del-CFTR correction, which make the hit optimization campaign challenging. Further evidence is therefore required in order to gain in-depth knowledge about the therapeutic relevance of these new approaches and to anticipate possible side effects. Even those proteins acting on CFTR trafficking whose structures or binding sites are unknown represent CF drug discovery issues, as they make novel small molecules difficult to design.

While preparing this paper, we also noted that the suitability of the molecular target chosen is an important point of discussion in proteostasis regulator research. Many studies focused on targets found in cell models that were different from human airway cells, and for this reason, sometimes they failed to rescue mutant CFTR when tested in primary bronchial epithelial cell models. This typically happens because there might be differences in the QC mechanisms between cell lines and primary cell models. Thus, validation of novel corrector compounds in well-differentiated airway cells appears mandatory in order to have meaningful data of efficacy that could guide the drug discovery process and, more importantly, to prioritize those whose mechanism of action is more effective on disease-relevant cell models.

**Figure 26:**
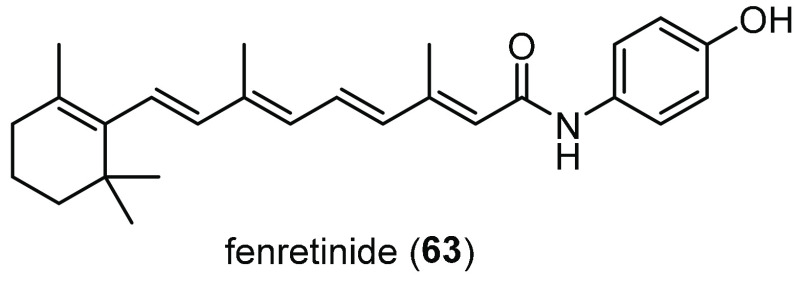
Chemical structure of fenretinide (**63**).

As shown throughout this review, a variety of mechanisms have been proposed to further enhance CFTR folding, trafficking, and function in a variety of systems and cell lines. However, the number of druggable targets belonging to the CFTR interactome and regulome that remain uninvestigated is high. To develop more effective drugs, a more complete understanding of them is necessary. For example, the interactions between CFTR and the PM it resides in has not been extensively explored. Lipids are known to have a large impact on other ABC transporters’ stability and function.^244^ Therefore, it is very likely that lipids modulate the function and stability of CFTR, too. It is well-documented that CF epithelial cells have an imbalance in the levels of polyunsaturated fatty acids (PUFAs), with reduced levels of DHA and increased levels of arachidonic acid (AA), which could lead to a proinflammatory state.^245−247^ CF cells also show increased levels of ceramide and glucosylceramide,^248−251^ which may contribute to chronic inflammation and increased susceptibility to *P. aeruginosa* seen in CF patients.^252,253^ Furthermore, CF cells expressing mutated CFTR channels are characterized by a decrease in glycosphingolipid GM1 content, which results in a diminished capacity for cell wound repair after injury.^254,255^ Mancini et al. recently demonstrated that the recovery of GM1 PM levels by its exogenous administration could stabilize the rescued F508del-CFTR protein and its PM interactome, leading to a significant improvement in the chloride transport of the mutant channel in association with **1** and **2**.^255^ From these data, it appears that the CFTR protein needs a proper organization of the PM environment to exert its function, and that GM1 could play a key role therein. Restoring the PM proper composition is fundamental for CFTR stability and function. Indeed, Garić et al. demonstrated that the proinflammatory imbalance in fatty acids could be causally linked to the lack of functional CFTR at the PM, as the correction of CFTR protein deficiency normalized the imbalance among ceramide species.^249^ The authors also demonstrated that fenretinide (**63**, Figure 26), a synthetic analogue of retinoic acid in study for cancer therapy, could rebalance ceramide levels by down-regulating ceramide synthase 5 (Cers5) enzyme activity.^249^ In CFTR knockout mice, **63** decreased AA levels and simultaneously increased DHA levels, thus efficiently correcting the proinflammatory lipid imbalance.^247^ Additionally, **63** treatment significantly decreased bacterial burden upon infection of CF mice with *P. aeruginosa*.^248^ Due to its pleiotropic effects, the underlying mechanism of **63** is still unclear. Likewise, the mechanistic connection between lipid modulation by **63** and CFTR expression and function remains unexplored. Nevertheless, this evidence suggests that only a fine coordination of PM interactome creates the proper PM environment for innate immunity, host defense, CFTR stability, and activity, thus opening a new scenario toward developing new alternative treatments for CF.

## 9 Future Prospects and Final Thoughts

CF is the most frequent among the autosomal recessive, lethal rare genetic diseases. Types and severity of symptoms can differ widely from person to person. While the CFTR genotype correlates with pancreatic status, the correlations between the CFTR genotype and lung and gastrointestinal phenotypes are debated, with CF patients bearing the same genotype displaying heterogeneous phenotypes.^256^ Over the last 30 years, advances in specialized CF care and in the CF therapeutic landscape have increased the expectancy and quality of life of people with CF. Industry and academic institutions have done extensive work to develop efficient CFTR potentiator and corrector molecules, as approximately 80% of CF patients can now benefit from one or more approved drugs. However, these therapies can only afford a partial rescue of the mutant CFTR channel,^40^ and their cost remains very high, leading to accessibility disparities worldwide. While for the F508del mutation, the efficacy of current corrector compounds, although partial, may be sufficient to achieve a marked clinical benefit,^35,257^ other mutations, displaying defective processing and trafficking to the PM, still lack effective therapies. In this way, proteostasis regulators shall have a wide applicability, as they target specific steps in CFTR processing that may create bottlenecks in its rescue. In addition, by addressing different cellular pathways, proteostasis regulators are likely to be useful tools to target protein trafficking diseases other than CF. For that, this innovative strategy holds great promise, but its application still has a long way to go. The way in which proteostasis regulators are implemented will be key to their success. First, it must be accepted that these compounds have effectiveness only in combination with other CFTR rescuing modulators. Indeed, cellular QC mechanisms are functionally redundant, and the modulation of one of those elements may not have a significant effect on the global biological outcome due to adaptive responses. In this way, proteostasis regulators alone will probably never reach levels of therapeutic efficacy equal to currently approved corrector/potentiator combinations. If a proteostasis regulator is to be developed, one must consider the greater potential for side effects of its underlying mechanism, as all the biological pathways involved in CFTR processing are interconnected. Alternatively, if proteostasis regulator compounds are implemented to specifically target those pathways that create the main bottlenecks in CFTR rescue, it is possible that through their combination with current therapies the dosage and therefore side effects of these treatments could be lowered. Additionally, many proteostasis regulators have shown positive secondary effects along with CFTR rescue activity, such as anti-inflammatory or innate host defense stimulatory effects against invading bacteria.^199,258^ Those pleiotropic effects, if well-calibrated, could have a great impact on CF patients suffering from chronic lung infections and inflammation. With these considerations in place, the future of proteostasis regulators could be bright, new research projects involved in drug-like molecules discovery will grow, and hopefully, this will be reflected into the clinic.

Future research should employ new cellular models that best represent CF disease and that can best mimic a given patient response to different treatments. This includes two-dimensional primary cultures from CF patients’ nasal brushing and three-dimensional organoids from airway or intestinal cells.^259−261^ Concerning the high costs and difficulties associated with drug development for this orphan disease, drug-repositioning technology may represent an auxiliary option to reduce both costs and likelihood of adverse effects. However, this field has to be considered complementary to up-to-date CF drug discovery pipelines. In this regard, the use of novel bioinformatics virtual screening protocols, such as high-performance computing (HPC), will allow the handling of large libraries of chemical compounds for new CFTR-rescuing hit identification, as recently seen for other diseases.^262^ Additionally, machine learning algorithm and artificial intelligence shall be exploited to speed up the extraction of results from omics data libraries and to power chemical synthetic processes.^263^

Given the need to develop novel CF treatment options, cross-disciplinary communication appears vital. CF foundations, companies, and academic laboratories have to find new ways to exchange knowledge and collaborate in order to encourage the exploration of previously uninvestigated targets. This will be fundamental for the expansion of the pioneering approach of CFTR proteostasis regulators in the future, which confidently will lead to new effective therapies for CF.

## Abbreviations

2-Nal: 3-(2-naphthyl)-l-alanine
AA: arachidonic acid
Aha1: activator of the Hsp90 ATPase
AS: AlphaScreen
Az: Apoptozole
BAG3: Bcl2-associated athanogene 3
BHK: baby hamster kidney cells
Bta: 3-(3-benzothienyl)-l-alanine
CaCC: calcium activated chloride channel
CAL: CFTR-associated ligand
CALP: CAL PDZ domain
Cers5: ceramide synthase 5
CF: cystic fibrosis
CFBE: bronchial epithelial cells
CFTR: cystic fibrosis transmembrane conductance regulator
CHIP: C-terminus of Hsc70-interacting protein
CK2: casein kinase 2
CME: clathrin-mediated endocytosis
CPP: cell permeating peptide
DHA: docosahexaenoic acid
DUBs: deubiquitinating enzymes
DUBTAC: deubiquitinase-targeting chimera
EGCG: epigallocatechin-3-gallate
ER: endoplasmic reticulum
ERAD: endoplasmic reticulum associated degradation
ERQC: endoplasmic reticulum quality control
esiRNA: endoribonuclease-prepared short interfering RNA
FA: fluorescence anisotropy
FAP: fluorogen-activating-protein
FICT: fluorescein isothiocyanate
FMP: FLIPR membrane potential assay
FP: fluorescence polarization
FRT: Fischer rat thyroid cells
GSH: glutathione
HATs: histone acetyl transferases
HBE: primary human bronchial epithelial cells
HDACs: histone deacetylases
HDACi: histone deacetylase inhibitors
Hsc: heat shock cognate
Hsp: heat shock protein
HS-YFP: halide-sensitive yellow fluorescent protein
IDO1: indoleamine 2,3-dioxygenase 1
NEF: nucleotide exchange factors
NHERF: Na^+^/H^+^ exchanger regulatory factors
NMD: nonsense-mediated mRNA decay
PAINS: pan-interference compounds
PARP: poly(ADPribose) polymerase
PARPi: PARP inhibitor
PCBP: poly-r(C) binding protein
PDB: protein data bank
Pen: l-penicillamine
Pip: pipecolic acid
PM: plasma membrane
ppFEV_1_: percent predicted forced expiratory volume in 1 s
PPI: protein protein interaction
PROTACs: proteolitically targeting chimeras
PUFA: polyunsaturated fatty acid
RNF5: ring finger protein 5
SAHA: suberoylanilid hydroxamic acid
SGC: sulfagalactosylceramide
SGL: sulfagalactolipid
siRNA: small interfering RNA
SRP: signal recognition particle
TG2: transglutaminase 2
Tle: *tert*-butyl-l-alanine
TPD: targeted protein degradation
TPS: targeted protein stabilization
Tα1: thymosin alpha 1
Ub: ubiquitin
UPR: unfolded protein response
UPS: ubiquitin-proteasome system
F508del: deletion of phenylalanine 508
